# Two New Lyngbyatoxin Derivatives from the Cyanobacterium, *Moorea producens*

**DOI:** 10.3390/md12125788

**Published:** 2014-12-01

**Authors:** Weina Jiang, Satoshi Tan, Yusuke Hanaki, Kazuhiro Irie, Hajime Uchida, Ryuichi Watanabe, Toshiyuki Suzuki, Bryan Sakamoto, Michiya Kamio, Hiroshi Nagai

**Affiliations:** 1 Department of Ocean Sciences, Tokyo University of Marine Science and Technology, Tokyo 108-8477, Japan; E-Mails: jwnkouina@gmail.com (W.J.); sa19901-31@gc4.so-net.ne.jp (S.T.); huchida.msr181823@gmail.com (H.U.); mkamio@kaiyodai.ac.jp (M.K.); 2 Division of Food Science and Biotechnology, Graduate School of Agriculture, Kyoto University, Kyoto 606-8502, Japan; E-Mails: y_hanaki_0625@yahoo.co.jp (Y.H.); irie@kais.kyoto-u.ac.jp (K.I.); 3 National Research Institute of Fisheries Science, Yokohama 236-8648, Japan; E-Mails: rwatanabe@affrc.go.jp (R.W.); tsuzuki@affrc.go.jp (T.S.); 4 Richard L. Roudebush VA Medical Center, Indianapolis, IN 46202, USA; E-Mail: bryan.sakamoto@va.gov

**Keywords:** lyngbyatoxin A, cyanobacteria, *Moorea producens*, toxicity, protein kinase C

## Abstract

The toxin-producing cyanobacterium, *Moorea producens*, is a known causative organism of food poisoning and seaweed dermatitis (also known as “swimmer’s itch”). Two new toxic compounds were isolated and structurally elucidated from an ethyl acetate extract of *M. producens* collected from Hawaii. Analyses of HR-ESI-MS and NMR spectroscopies, as well as optical rotations and CD spectra indicated two new lyngbyatoxin derivatives, 2-oxo-3(*R*)-hydroxy-lyngbyatoxin A (**1**) and 2-oxo-3(*R*)-hydroxy-13-*N*-desmethyl-lyngbyatoxin A (**2**). The cytotoxicity and lethal activities of **1** and **2** were approximately 10- to 150-times less potent than lyngbyatoxin A. Additionally, the binding activities of **1** and **2** possessed 10,000-times lower affinity for the protein kinase Cδ (PKCδ)-C1B peptide when compared to lyngbyatoxin A. These findings suggest that these new lyngbyatoxin derivatives may mediate their acute toxicities through a non-PKC activation pathway.

## 1. Introduction

Cyanobacteria, often called “blue-green algae”, have a long evolutionary and biochemical history [1]. Some cyanobacteria have been found to cause hepatic, neurologic and dermal system damage due to the “cyanotoxins” they produce [2]. One of the most toxic genera of filamentous marine cyanobacteria, *Moorea producens* (formerly classified as *Lyngbya majuscula*) [3], is a rich source of diverse compounds that possess a variety of biological activities [4,5,6]. Aplysiatoxin, debromoaplysiatoxin and lyngbyatoxin A are produced by *M. producens* and have been reported to be the causative agents of seaweed dermatitis (also known as “swimmer’s itch”) [7,8,9]. The first reported case of seaweed dermatitis was from Hawaii in 1958. On the island of Oahu, Hawaii, more than 125 people were reported to have dermatitis symptoms secondary to exposure to *M. producens* [10,11]. To date, numerous incidences of seaweed dermatitis (skin, eye and respiratory irritation) have been reported from the Pacific region [12]. Additionally, aplysiatoxin, debromoaplysiatoxin and lyngbyatoxin A are known compounds that can cause food poisoning. Symptoms of diarrhea, vomiting and skin ulcers from the consumption of meat from the marine turtle, *Chelonia mydas*, have been reported [13,14]. Lyngbyatoxin A was identified to be the cause of this intoxication [15]. In Hawaii, the ingestion of the red alga, *Gracilaria coronopifolia*, was reported to induce symptoms of food poisoning [16,17,18]. The characteristic symptoms of this intoxication were vomiting, diarrhea and a burning sensation of the mouth and throat [16]. Aplysiatoxin and its related compounds were identified as the causative agents in these toxic events [17,19,20,21]. The cyanobacteria-like organism, *G. coronopifolia*, was reported to be the true origin of these toxins [17]. Toxins from *M. producens* are known to possess potent tumor-promoting activity, which can induce tumor formation [22,23]. Lyngbyatoxin A is a known potent tumor promoter by its ability to strongly induce protein kinase C (PKC) activity [22,24].

**Figure 1:**
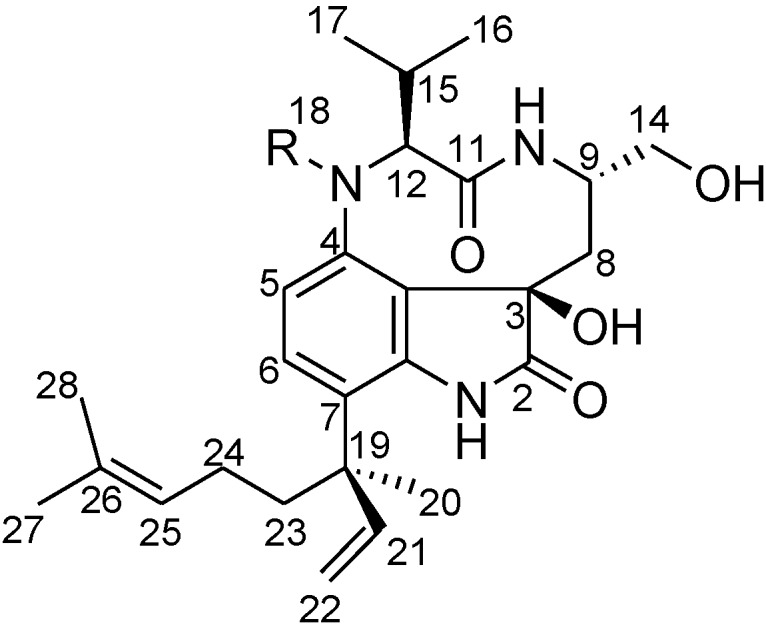
Structures of **1** and **2**.

**1**: 2-oxo-3(*R*)-hydroxy-lyngbyatoxin A, R: Me; **2**: 2-oxo-3(*R*)-hydroxy-13-*N*-desmethyl-lyngbyatoxin A, R: H.

**Figure 2:**
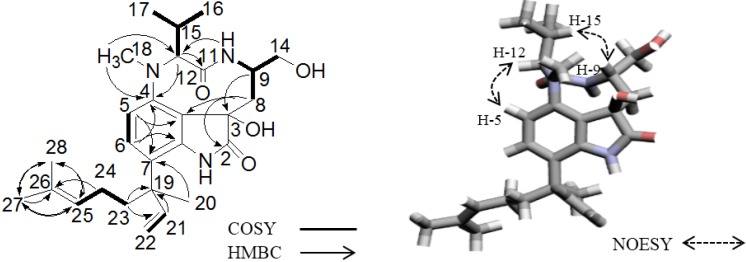
The structure of **1** and its key 2D NMR correlations in CD_3_OD.

The isolation, structural elucidation and biological activities of two novel lyngbyatoxin derivatives, 2-oxo-3(*R*)-hydroxy-lyngbyatoxin A (**1**, Figure 1) and 2-oxo-3(*R*)-hydroxy-13-*N*-desmethyl-lyngbyatoxin A (**2**, Figure 1), from *M. producens* are presented.

## 2. Results and Discussion

### 2.1. Isolation of **1** and **2**

After extraction of the freeze-dried sample, the crude extracts were evaporated to a dried residue. Liquid-liquid partition was then performed on the residue. The EtOAc fraction possessed the most potent activity determined by cytotoxic and crustacean lethal assays. This fraction was subjected to an open glass column for ODS chromatography and fractionated with various methanol solutions (50%, 70%, 90% and 100% methanol). The seventy percent methanol fraction was further purified using a reversed phase HPLC and recycling ODS HPLC system. Two pale yellow solid compounds, **1** (1.37 mg, 0.09%) and **2** (1.96 mg, 0.13%), were isolated, and the measurement of proton NMR spectra further certified the purity of the two newly-isolated compounds.

### 2.2. Elucidation of the Planar Structures of **1** and **2**

Compound **1** possessed a molecular formula of C_27_H_39_N_3_O_4_ suggested by HR-ESI-MS according to the [M + H]^+^ ion peak at *m/z* 470.3051 (calcd. for C_27_H_40_N_3_O_4_, 470.3019) and the [M + Na]^+^ ion peak at *m/z* 492.2873 (calcd. for C_27_H_39_N_3_O_4_Na, 492.2838). A molecular formula of C_26_H_37_N_3_O_4_ was suggested for **2** based on the mass spectrum at *m/z* 456.2901 [M + H]^+^ (calcd. for C_26_H_38_N_3_O_4_, 456.2862), which had one less methyl or methylene moiety when compared to **1**. The UV spectral data of **1** and **2** suggested the existence of a conjugated ring system (**1**, UV λmax (EtOH) nm (log ε), 239 (4.19), 284 (3.71); **2**, UV λmax (EtOH) nm (log ε), 240 (4.54), 287 (3.73)).

The structure of **1** was predominantly determined by 1D and 2D NMR spectral analyses. The tabulated ^13^C and ^1^H NMR spectral data for **1** are shown in Table 1. The ^1^H NMR spectrum of **1** resembled lyngbyatoxin A. Comparing the ^1^H NMR spectrum of **1** in methanol-*d*_4_ (CD_3_OD) with that of lyngbyatoxin A [8], all of the proton signals in lyngbyatoxin A were observed, except for the signal corresponding to H-2. While lyngbyatoxin A shows an olefinic carbon at C-2 (δ_C_ 120.7) and C-3 (δ_C_ 114.1) from the ^13^C NMR spectral data of lyngbyatoxin A, the ^13^C NMR spectral data of **1** revealed a carbonyl carbon at C-2 (δ_C_ 182.1) and a quaternary carbon at C-3 (δ_C_ 76.0), indicating the existence of an oxindole moiety in **1**. This was also supported by the UV spectra [25]. The correlations observed in the ^1^H-^1^H COSY and ^1^H–^13^C HMBC spectra of **1** are shown in Figure 2. From these results, **1** was inferred to be 2-oxo-3-hydroxy-lyngbyatoxin A (Figure 2, left). Furthermore, **2** lacked a C-18 *N*-methyl group from the data analysis of the 1D and 2D NMR spectra, indicating that **2** was 2-oxo-3-hydroxy-13-*N*-desmethyl-lyngbyatoxin A. The deduced planar structures were also supported by the close similarity of the ^1^H NMR spectral data of 2-oxo-teleocidin A-1 [26].

**Table 1:** ^1^H and ^13^C NMR chemical shifts observed for **1** and **2** in CD_3_OD.

No.	1		2	
	^13^C	^1^H (mult, *J* in Hz)	^13^C	^1^H (mult, *J* in Hz)
2	182.1		181.5	
3	76.0		75.9	
3a	128.6		122.5	
4	151.0		145.6	
5	120.0	7.15, 1H (d, 8.6)	116.9	6.69, 1H (d, 8.6)
6	130.2	7.29, 1H (d, 8.6)	129.8	7.15, 1H (d, 8.6)
7	128.3		123.7	
7a	139.7		138.8	
8	42.4	2.24, 1H (d, 15.1)	42.9	2.15, 1H (m)
		1.46, 1H (dd, 9.0, 15.1)		1.50, 1H (dd, 9.4, 14.7)
9	51.1	5.63, 1H (m)	51.6	4.49, 1H (m)
11	175.1		176.4	
12	78.1	3.81, 1H (d, 5.5)	74.3	3.78, 1H (d, 10.5)
14	67.5	3.60, 1H (dd, 4.7, 10.9)	67.1	3.54, 1H (dd, 5.0, 11.1)
		3.54, 1H (dd, 5.6, 10.9)		3.49, 1H (dd, 7.2, 11.1)
15	30.0	2.43, 1H (m)	32.6	2.15, 1H (m)
16	22.7	1.22, 3H (d, 6.9)	21.3	1.29, 3H (d, 6.5)
17	19.4	1.20, 3H (d, 6.9)	20.6	1.13, 3H (d, 6.5)
18	42.3	2.67, 3H (s)		
19	44.6		44.4	
20	25.4	1.41, 3H (s)	25.6	1.38, 3H (s)
21	146.8	6.09, 1H (dd, 10.7, 17.6)	147.2	6.08, 1H (dd, 10.7, 17.6)
22	114.0	5.22, 1H (d, 10.7)	113.6	5.19, 1H (d, 10.7)
		5.06, 1H (d, 17.7)		5.04, 1H (d, 17.7)
23	40.2	1.86, 1H (m)	40.5	1.83, 1H (m)
		1.76, 1H (m)		1.72, 1H (m)
24	24.2	1.97, 1H (m)	24.3	1.94, 1H (m)
		1.76, 1H (m)		1.74, 1H (m)
25	125.3	5.10, 1H (t, 5.8)	125.4	5.11, 1H (m)
26	132.5		132.4	
27	25.8	1.68, 3H (s)	25.8	1.67, 3H (s)
28	17.7	1.55, 3H (s)	17.7	1.55, 3H (s)

s, singlet; d, doublet; t, triplet; m, multiplet.

### 2.3. Elucidation of Absolute Stereochemistry of **1** and **2**

**Figure 3:**
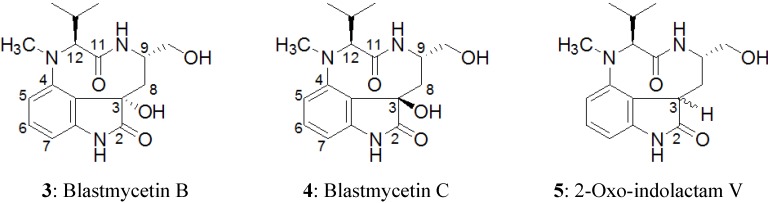
Structures of blastmycetin B (**3**), blastmycetin C (**4**) and 2-oxo-indolactam V (**5**).

The NOESY correlations (H-5/H-12 and H-9/H-15) of **1** indicated that the N-10/C-11 amide bond was *cis* by molecular model-building. One dominant conformer (*cis* amide conformer) was observed in the ^1^H NMR spectrum of **1**, similar to lyngbyatoxin A. Therefore, the relative stereochemistry at C-9 and C-12 was suggested to be 9S*, 12S* (Figure 2, right). It was also deduced that **2** had 9S*, 12S* relative stereochemistry from the results of NOESY correlations. Blastmycetins B (**3**, Figure 3) and C (**4**, Figure 3) were previously isolated from the culture broth of the actinomycete, *Streptoverticillium blastmyceticum* [27]. The planar structures of **3** and **4** were a partial structure of **1**. Furthermore, **3** and **4** had a 9*S*, 12*S* configuration. The only structural difference between **3** and **4** was the configuration at the C-3 hydroxyl group. Therefore, **3** and **4** could be the model compounds for the determination of the configuration on C-3 of **1** and **2**. The reported proton chemical shifts were 2.05, 2.17 (H-8), 3.95 (H-9) and 5.96 (H-12) for **3** and 1.46, 2.22 (H-8), 5.61 (H-9) and 3.76 (H-12) for **4** [25]. The ^1^H chemical shifts obtained in this study were 1.46, 2.24 (H-8), 5.63 (H-9) and 3.81 (H-12) for **1** and 1.50, 2.15 (H-8), 4.49 (H-9) and 3.78 (H-12) for **2** (Table 1). The results indicated that both **1** and **2** had a 3R*, 9S*, 12S* relative stereochemistry. However, the downfield shift of H-9 (δ_H_ 4.49) of **2** was not so remarkable compared to the H-9 (δ_H_ 5.63) of **1**. This might be caused by the lack of a C-18 *N*-methyl group in **2**, leading to the conformational change of **2**. To further analyze the configuration of **2**, NMR spectra of **2** were measured in chloroform-*d* (see Supplementary Information). The ^1^H NMR spectra showed protons of two hydroxyl groups at C-3 and C-14 with chemical shifts of δ_H_ 4.36 and δ_H_ 3.37, respectively. Moreover, the NOESY spectra showed NOE correlations between H-9/H-13, H-14 and H-15, OH-3/H-13 and OH-14, H-8 (δ2.13, d)/H-14' and H-8' (δ1.59, dd)/H-10. These observations finally confirmed that **2** had the relative stereochemistry of 3R*, 9S*, 12S*.

Furthermore, compound **3** showed levorotatory optical rotation, while **4** showed dextrorotatory optical rotation [25]. Both **1** and **2** showed dextrorotatory optical rotation (**1**, ${\left[\text{α}\right]}_{\text{D}}^{24}$ +119.2° (c 0.18, CH_3_OH); **2**, ${\left[\text{α}\right]}_{\text{D}}^{24}$ +150.5° (c 0.30, CH_3_OH)), which was the same as that of **4**. Therefore, the absolute configurations of both **1** and **2** were deduced to be 3*R*, 9*S*, 12*S*.

**Figure 4:**
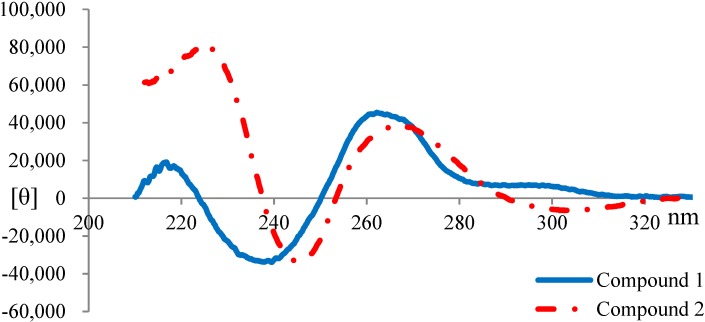
CD spectra of **1** and **2**.

The inference in the absolute configurations on C-19 of the linalyl group in lyngbyatoxin A derivatives was elucidated by the analysis of the circular dichroism (CD) spectra at around 230 nm. The upward and downward curves around 230 nm indicated the absolute configurations to be 19-*R* and 19-*S*, respectively [28,29,30]. The CD spectra of **1** and **2** are shown in Figure 4. Both compounds showed upward curves around 230 nm, indicating that **1** and **2** had the same absolute configuration *R* at C-19. Therefore, it was deduced that both **1** and **2** had 3*R*, 9*S*, 12*S*, 19*R* absolute configurations. Consequently, **1** was 2-oxo-3(*R*)-hydroxy-lyngbyatoxin A and **2** was 2-oxo-3(*R*)-hydroxy-13-*N*-desmethyl-lyngbyatoxin A (Figure 1).

While compounds containing 2-oxo-indolactam V (**5**, Figure 3) have been isolated from actinomycetes [25,26], this is the first report describing 2-oxo derivatives of lyngbyatoxin A from the cyanobacterium, *M. producens*.

The LC-MS/MS analyses of the 70% methanol fraction were carried out to detect the existence of isomers of **1** and **2**. The existence of another compound, which had the same molecular weight and MS/MS pattern with **1**, was detected. Since **3** and **4** had been isolated from the same actinomycete species [27], this new unidentified compound might be 2-oxo-3(*S*)-hydroxy-lyngbyatoxin A. Unfortunately, we were unable to isolate this compound. While an isomer of **2** could not be detected by LC-MS/MS, 2-oxo-3(*S*)-hydroxy-13-*N*-desmethyl-lyngbyatoxin A may not have been present in the sample.

### 2.4. Biological Activity Tests

The effects of two lyngbyatoxin A derivatives, **1** and **2**, on the inhibition of cell proliferation were determined using L1210 leukemia cells. The IC_50_ values determined for **1** and **2** were 98 and 321 μM, respectively. Recent reports indicated that the IC_50_ value of lyngbyatoxin A against L1210 cells was 8.1 μM [30]. The cytotoxic activities of **1** and **2** were approximately 10- to 40-times less potent than lyngbyatoxin A.

Crustacean lethal activity tests were performed using the shrimp, *Palaemon paucidens*. Injection of **1** and **2** caused paralysis of the tested shrimp in the first 30 min of exposure. The lethal dose 33% values (LD_33_) were used to estimate the lethal toxicity. While the LD_33_ values of **1** and **2** were 89 and 25 mg/kg, respectively, the LD_33_ value of lyngbyatoxin A was 0.6 mg/kg. The lethal activities of **1** and **2** were approximately 150- and 40-times less potent than lyngbyatoxin A, respectively.

Lyngbyatoxin A is a potent carcinogen by acting as a powerful activator of all conventional and novel PKC isozymes (PKCs) secondary to its direct binding of the C1 domain [22,23,31,32]. Since PKCδ is an important PKCs involved in carcinogenesis [33], the binding affinities of PKCδ were tested using a synthetic PKCδ-C1B peptide by a competitive binding assay with [^3^*H*]phorbol 12,13-dibutyrate (PDBu) [34]. The *K_i_* value for the inhibition of [^3^*H*]PDBu-binding with lyngbyatoxin A is 0.11 nM [30]. In this study, it was shown that **1** and **2** had *K_i_* values of 1400 and 940 nM, respectively.

The cytotoxicity and lethal activities of **1** and **2** were approximately 10- to 150-times less potent than lyngbyatoxin A. Additionally, the binding activities of **1** and **2** possessed 10,000-times lower affinity for the protein kinase Cδ (PKCδ)-C1B peptide when compared to lyngbyatoxin A. These results might be simply caused by the difference in bioavailability or localization of these new compounds and lyngbyatoxin A in the different assays. However, in the previous biological activity tests, 12-*epi*-lyngbyatoxin A and lyngbyatoxin A showed comparable acute toxicities, but 12-*epi*-lyngbyatoxin A had more than 100-times lower binding affinity for PKCδ compared to lyngbyatoxin A [30]. Therefore, we had proposed the hypothesis that the acute toxicities (cytotoxicity and lethality) of lyngbyatoxin-type compounds might be mediated through a non-PKC activation pathway [30]. In this study, acute toxicities (cytotoxicity and lethal activities) versus PKCδ binding activities of **1** and **2** showed big difference with those of lyngbyatoxin A. Thus, we considered that the results obtained in this study probably would also support the hypothesis we proposed and an alternate target instead of PKC isozymes might exist for the acute toxicities of these toxins.

Therefore, further studies are necessary to verify our proposed hypothesis, as well as to elucidate whether there exist other receptors in relation to the acute toxicities of lyngbyatoxin-type compounds.

*M. producens* producing lyngbyatoxin A, aplysiatoxin and their related compounds have been reported to be the causative agents of swimmer’s itch and food poisoning [8,14,15]. New Compounds **1** and **2** showed acute toxicity, albeit weak; thus, we might pay attention to these compounds also on health hazard cases involving *M. producens*.

## 3. Experimental Section

### 3.1. Instrumentation and General Methods

Optical rotations were measured on a JASCO P-2100 (JASCO Co., Tokyo, Japan). Circular dichroism (CD) spectra were measured on a JASCO J-715 (JASCO Co., Tokyo, Japan). UV spectra were measured on a HITACHI U-3000 (Hitachi High-Tech Fielding Co., Tokyo, Japan). NMR spectra were recorded on Bruker AVANCE III 800 MHz and 600 MHz spectrometers (Bruker Co., Bremen, Germany). HR-ESI-MS spectra data were recorded on a Bruker micrOTOF QII (Bruker Co., Bremen, Germany) mass spectrometer. LC-MS/MS analyses were performed using an Ultimate 3000 liquid chromatograph (Thermo Dionex, Sunnyvale, CA, USA), which was coupled to a Bruker micrOTOF QII (Bruker Co., Bremen, Germany) mass spectrometer. HPLC analyses were performed using a SHIMADZU HPLC system (SHIMADZU Co., Kyoto, Japan) equipped with a SPD-M10A diode array detector (SHIMADZU Co., Kyoto, Japan).

### 3.2. Sample Collection

The marine cyanobacterium, *M. producens*, was collected at Kahala Beach (Oahu, Hawaii) in 1998. The samples were freeze-dried immediately after the collection and stored in a freezer at −30 °C.

### 3.3. Extraction and Purification

A crude extract from the freeze-dried sample (823 g, dry weight) was prepared with organic solvents in sequence: ethanol, methanol and acetone. The three extracts were combined and evaporated. The residue (128 g, dry weight) was dissolved in 80% methanol and partitioned between hexane. After the 80% methanol layer was evaporated, the dried residue was re-suspended in distilled water and extracted with ethyl acetate (EtOAc). The EtOAc fraction (4.2 g, dry weight) was then separated by a 20 × 300 mm open glass column filled with ODS resin (Pegasil Prep ODS-7515-12A, Senshu Co., Tokyo, Japan) incorporating a stepwise increase in the methanol concentration (50%, 70%, 90% and 100% methanol). The various methanol solutions yielded the following: 50% methanol fraction (870 mg), 70% methanol fraction (1600 mg), 90% methanol fraction (1220 mg) and 100% methanol fraction (200 mg). From the 90% methanol fraction, we have previously isolated lyngbyatoxin A and 12-*epi*-lyngbyatoxin A [30]. In this study, reversed-phase high pressure liquid chromatography (HPLC) was utilized to further purify the 70% methanol fraction (1540 mg). The 70% methanol fraction was divided into 24 portions, and the HPLC was performed on each portion on a 20 × 250 mm column (SHISEIDO CAPCELL PAK C18, Shiseido Co., Tokyo, Japan) using a gradient elution system with the following conditions: a linear gradient from 75% to 100% methanol during the first 90 min, a flow rate of 3 mL/min and UV-Vis detection at 210, 230 and 270 nm. A recycle chromatography HPLC (system unit: Senshu Scientific SSC-1310, column: COSMOSIL C18-AR-II 10 × 250 mm, solvent: 73% MeOH for **1** and 72% MeOH for **2**, flow rate: 2 mL/min, detection: 210 nm) was performed for the isolation of **1** and **2**.

### 3.4. HR-ESI-MS and LC-MS/MS Analyses

The Bruker micrOTOF QII mass spectrometer was operated in both positive and negative ion modes. To detect the isomers of two newly-isolated compounds for LC-MS/MS analyses, a 2.0 × 250 mm column (Mightysil RP-18 GP, Kanto Chemical Co., Tokyo, Japan) was utilized. Eluent A was 0.1% formic acid in water and B was methanol-water (95:5), containing 0.1% formic acid. The mobile phase (25% A and 75% B) was isocratic with a flow rate of 0.2 mL/min.

### 3.5. Nuclear Magnetic Resonance Experiments

NMR assignment was achieved from 1D and 2D spectroscopies (^1^H, ^13^C, COSY, HSQC, HMBC and NOESY). Chemical shifts were reported in δ units (ppm) relative to CD_3_OD (δ_H_ at 4.87 ppm and δ_C_ at 49.0 ppm) and CDCl_3_ (δ_H_ at 7.26 ppm and δ_C_ at 77.2 ppm) solvents at 300 K.

### 3.6. Circular Dichroism (CD) Spectroscopy

The CD spectral data were recorded in methanol with the concentration of 213 μmol/L for **1** and 220 μmol/L for **2**, using a 2-mm path-length quartz cell. The measurements were performed at room temperature (25 °C). The spectra were measured from 380 nm to 195 nm, and a minimum of six scans was performed for each compound.

### 3.7. Cytotoxicity Assay

L1210 mouse leukemia cells were used for the cytotoxicity assay of **1** and **2**, as previously described [35]. Five microliters of each sample or methanol alone were placed into individual wells of a 96-well microtiter plate. The solvent was removed by air drying from the samples at room temperature on a clean bench. Five microliters of sterilized water were then applied to each well. After the samples were dissolved, 100 μL of the L1210 mouse leukemia cell solution (3 × 10^4^ cells/mL) were plated into individual wells of the 96-well microtiter plate. The assay plate was placed in an incubator for 24 h at 37 °C with 5% CO_2_. After heating the 2,3-bis-(2-methoxy-4-nitro-5-sulfophenyl)-2H-tetrazolium-5-carboxanilide inner sodium salt (XTT, Sigma, St. Louis, MO, USA) working solution to 37 °C, the solution was gently swirled until a clear solution was obtained. Fifty microliters of the XTT working solution were added into each well and then incubated for 6 hours at 37 °C with 5% CO_2_. The XTT working solution was prepared as a mixture of 5.5 mL of XTT in RPMI-1640 medium (1 mg/mL) and 170 μL of phenazine methosulfate (Sigma, St. Louis, MO, USA) in RPMI-1640 medium (38 μg/mL). The cells without sample treatment were used as a negative control. The inhibition of cell proliferation was determined by measuring the absorbance of each well via a microplate reader (Model 550, Bio-Rad, Hercules, CA, USA) at a wavelength of 450 nm.

### 3.8. Crustacean Lethality Test

The crustacean lethality of lyngbyatoxin A, **1** and **2** were tested using the shrimp, *Palaemon paucidens*, as previously described [30]. The average weight of the shrimp was 0.5 g. A lethal dose 33% value (LD_33_) was used to estimate the lethality of the compounds.

### 3.9. Binding Assay of PKC Ligands Using the PKCδ-C1B Peptide

Using previously-described methods, the inhibition of [^3^*H*]phorbol 12,13-dibutyrate (PDBu) binding to the protein kinase Cδ (PKCδ)-C1B peptide was evaluated [36,37]. The standard assay mixture contained: 13.8 nM PKCδ-C1B peptide, 20 nM [^3^H]PDBu (18.7 Ci/mmol, Perkin-Elmer Life Sciences, Waltham, MA), 50 μg/mL 1,2-dioleoyl-*sn*-glycero-3-phospho-l-serine (Sigma, St. Louis, MO, USA), 3 mg/mL bovine γ-globulin (Sigma, St. Louis, MO, USA) and various concentrations of inhibitors in a 50 mM Tris-maleate buffer (pH 7.4 at 4 °C). Total binding was measured in the absence of an inhibitor (only ethanol), and non-specific binding was evaluated in the absence of the PKCδ-C1B peptide. The concentration of the properly-folded PKCδ-C1B peptide was estimated to be about 3 nM on the basis of the *B*_max_ values from Scatchard analyses previously reported [36,37]. Binding affinity was evaluated as IC_50_ by the sample concentration required to cause 50% inhibition of the binding of [^3^*H*]PDBu to the PKCδ-C1B peptide [38]. The inhibition constant, *K_i_*, was calculated by the method of Sharkey and Blumberg [39].

## 4. Conclusions

In this study, two new toxic compounds were isolated and structurally elucidated from the Hawaiian cyanobacterium, *Moorea producens*. From the analyses of HR-ESI-MS and NMR spectroscopies, as well as optical rotations and CD spectra, these new compounds were indicated to be the new lyngbyatoxin derivatives, 2-oxo-3(*R*)-hydroxy-lyngbyatoxin A (**1**) and 2-oxo-3(*R*)-hydroxy-13-*N*-desmethyl-lyngbyatoxin A (**2**). The cytotoxicity and lethal activities of **1** and **2** were approximately 10- to 150-times less potent than lyngbyatoxin A. However, the PKCδ binding activities of **1** and **2** possessed 10,000-times lower affinity when compared to lyngbyatoxin A. These findings supported our hypothesis that acute toxicities, such as cytotoxicity, and lethality of these new lyngbyatoxin derivatives might be mediated through a non-PKC activation pathway.

## Supplementary Files

Supplementary File 1
