# Supplementary material for: Two New Lyngbyatoxin Derivatives from the Cyanobacterium, Moorea producens
Source: Mar Drugs. 2014 Dec 1;12(12):5788–800. doi: 10.3390/md12125788 (PMC4278201; doi:10.3390/md12125788)
Supplement: Supplementary File 1 [file marinedrugs-12-05788-s001.pdf]

Supplementary Information

Figure S1. Isolation and purification scheme of **1** and **2** from the Hawaiian cyanobacteria.

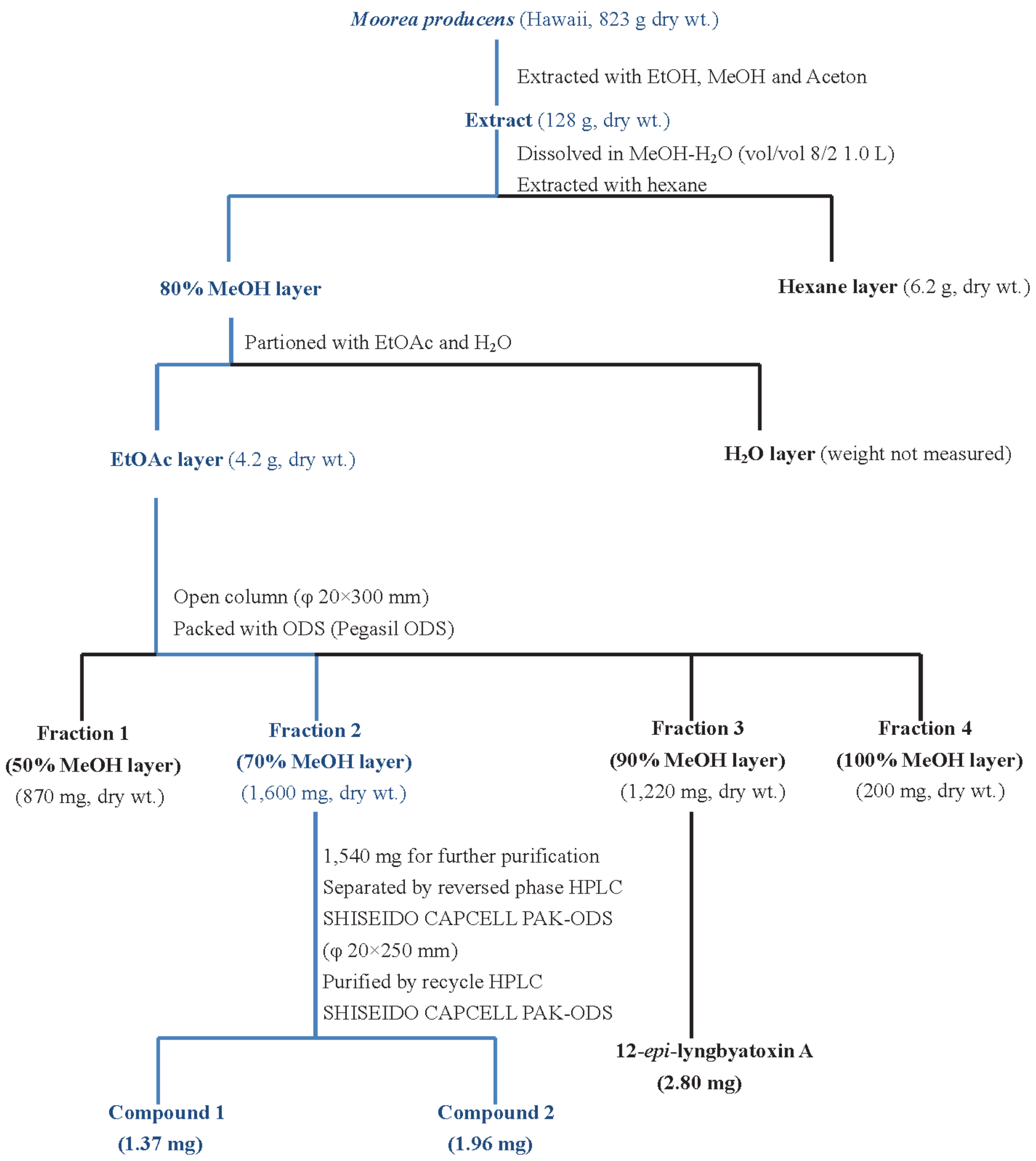

Figure S2. HR-ESI-MS spectrum of **1**.

Display Report

Analysis Info

Analysis Name	D:\Data\KOU\20140605_Kou\compound 1_1 ppm_GA1_01_1431.d	Acquisition Date	6/3/2014 9:23:56 PM
Method	20140603_1t_nega lcms.m	Operator	BDAL@DE
Sample Name	compound 1_1 ppm	Instrument	micrOTOF-Q 228888.10312
Comment			

Acquisition Parameter

Source Type	ESI	Ion Polarity	Negative	Set Nebulizer	1.6 Bar
Focus	Not active	Set Capillary	4200 V	Set Dry Heater	200 ° C
Scan Begin	300 m/z	Set End Plate Offset	-500 V	Set Dry Gas	8.0 l/min
Scan End	800 m/z	Set Collision Cell RF	450.0 Vpp	Set Divert Valve	Source

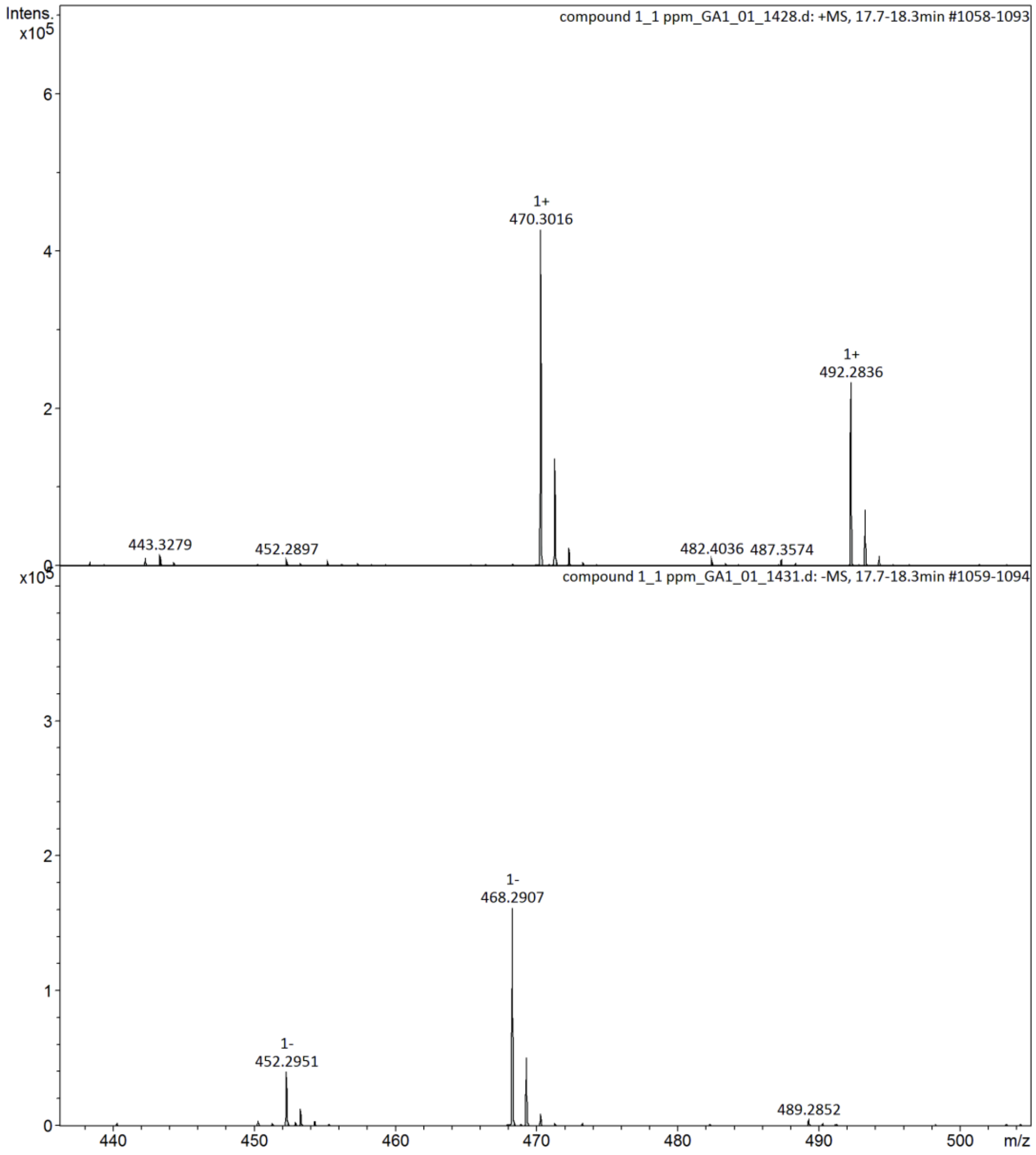

Bruker Compass DataAnalysis 4.1 printed:11/26/2014 6:18:13 PM by: KAMI O@DE Page 1 of 1

Figure S3. HR-ESI-MS spectrum of **2**.

Display Report

Analysis Info

Analysis Name	D:\Data\KOU\20140605_Kou\compound 2_1 ppm_GA2_01_1432.d	Acquisition Date	6/3/2014 10:10:49 PM
Method	20140603_lt_nega lcms.m	Operator	BDAL@DE
Sample Name	compound 2_1 ppm	Instrument	micrOTOF-Q 228888.10312
Comment			

Acquisition Parameter

Source Type	ESI	Ion Polarity	Negative	Set Nebulizer	1.6 Bar
Focus	Not active	Set Capillary	4200 V	Set Dry Heater	200 °C
Scan Begin	300 m/z	Set End Plate Offset	-500 V	Set Dry Gas	8.0 l/min
Scan End	800 m/z	Set Collision Cell RF	450.0 Vpp	Set Divert Valve	Source

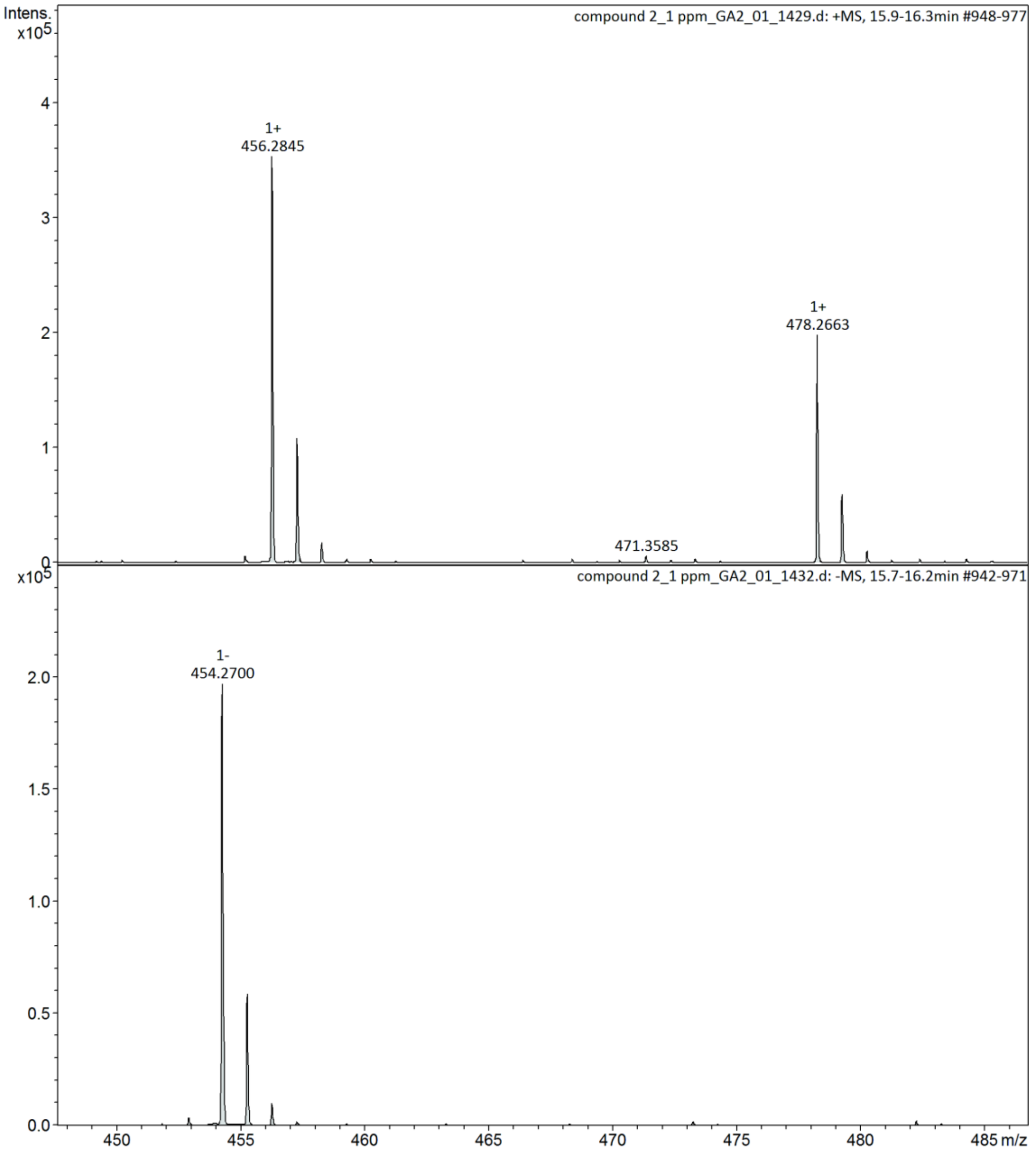

Figure S4. Negative LC-MS/MS spectra data of **1**.

Display Report

Analysis Info | Acquisition Date 6/4/2014 9:47:43 PM

Analysis Name	D:\Data\KOU\20140605_Kou\20140603_Moorea EtOAc_70%MeOH_GA3_01_1444.d		
Method	20140603_1t_nega lcmsms_468.3.m	Operator	BDAL@DE
Sample Name	20140603_Moorea EtOAc_70%MeOH	Instrument	micrOTOF-Q 228888.10312
Comment			

Acquisition Parameter

Source Type	ESI	Ion Polarity	Negative	Set Nebulizer	0.4 Bar
Focus	Not active	Set Capillary	3500 V	Set Dry Heater	180 ° C
Scan Begin	50 m/z	Set End Plate Offset	-500 V	Set Dry Gas	4.0 l/min
Scan End	3000 m/z	Set Collision Cell RF	150.0 Vpp	Set Divert Valve	Waste

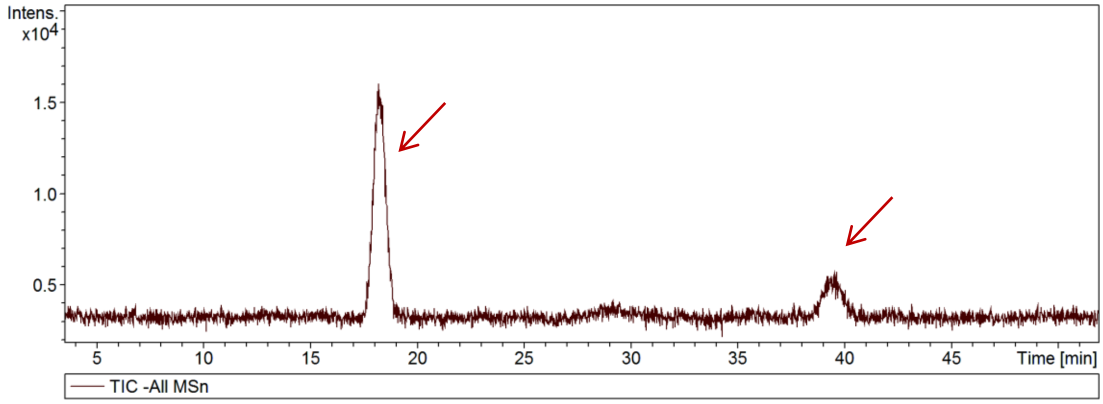

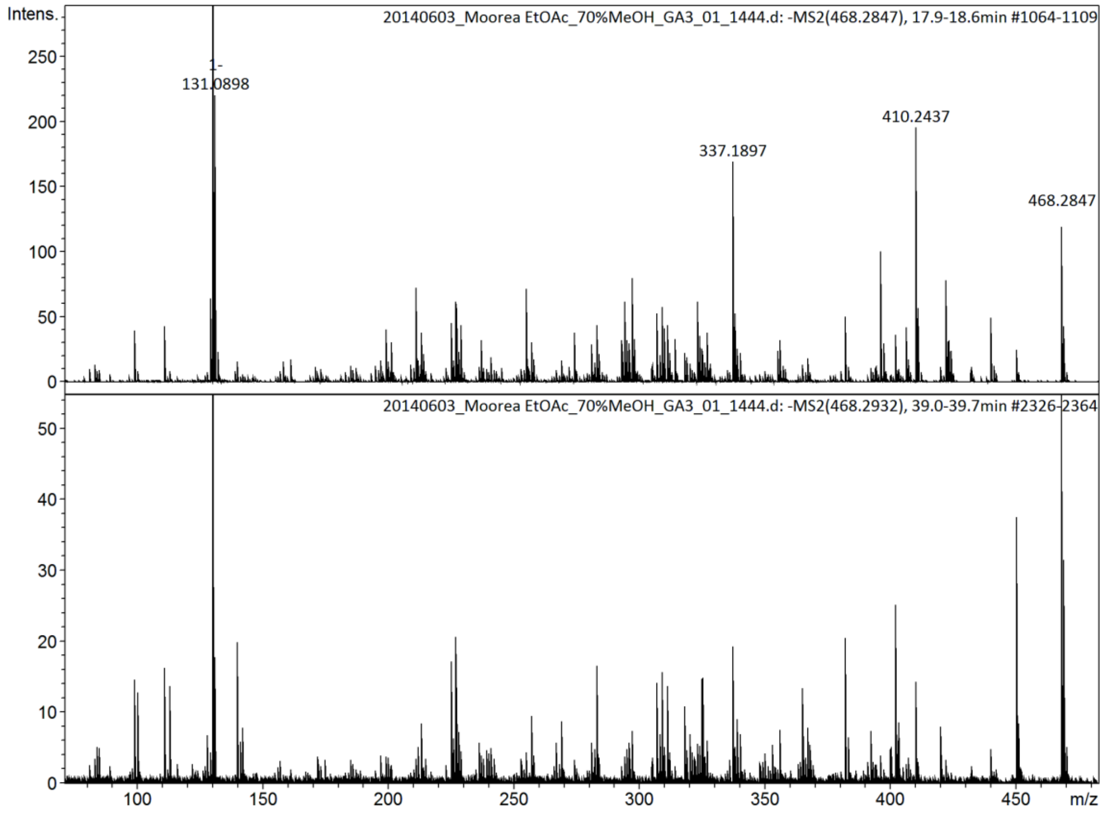

Figure S5. Negative LC-MS/MS spectra data of **2**.

Display Report

Analysis Info

Analysis Name	D:\Data\KOU\20140605_Kou\20140603_Moorea EtOAc_70%MeOH_GA3_01_1445.d	Acquisition Date	6/4/2014 10:49:40 PM
Method	20140603_lt_nega lcmsms_454.3.m	Operator	BDAL@DE
Sample Name	20140603_Moorea EtOAc_70%MeOH	Instrument	micrOTOF-Q 228888.10312
Comment			

Acquisition Parameter

Source Type	ESI	Ion Polarity	Negative	Set Nebulizer	0.4 Bar
Focus	Not active	Set Capillary	3500 V	Set Dry Heater	180 ° C
Scan Begin	50 m/z	Set End Plate Offset	-500 V	Set Dry Gas	4.0 l/min
Scan End	3000 m/z	Set Collision Cell RF	150.0 Vpp	Set Divert Valve	Waste

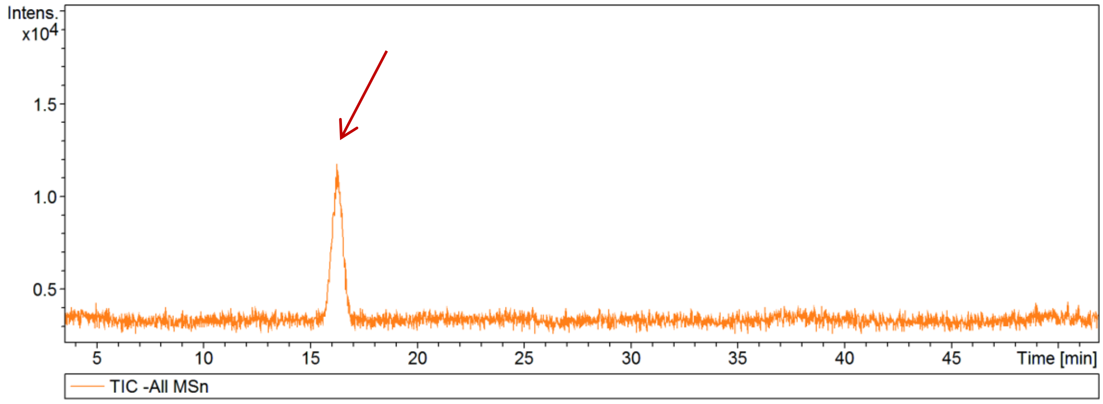

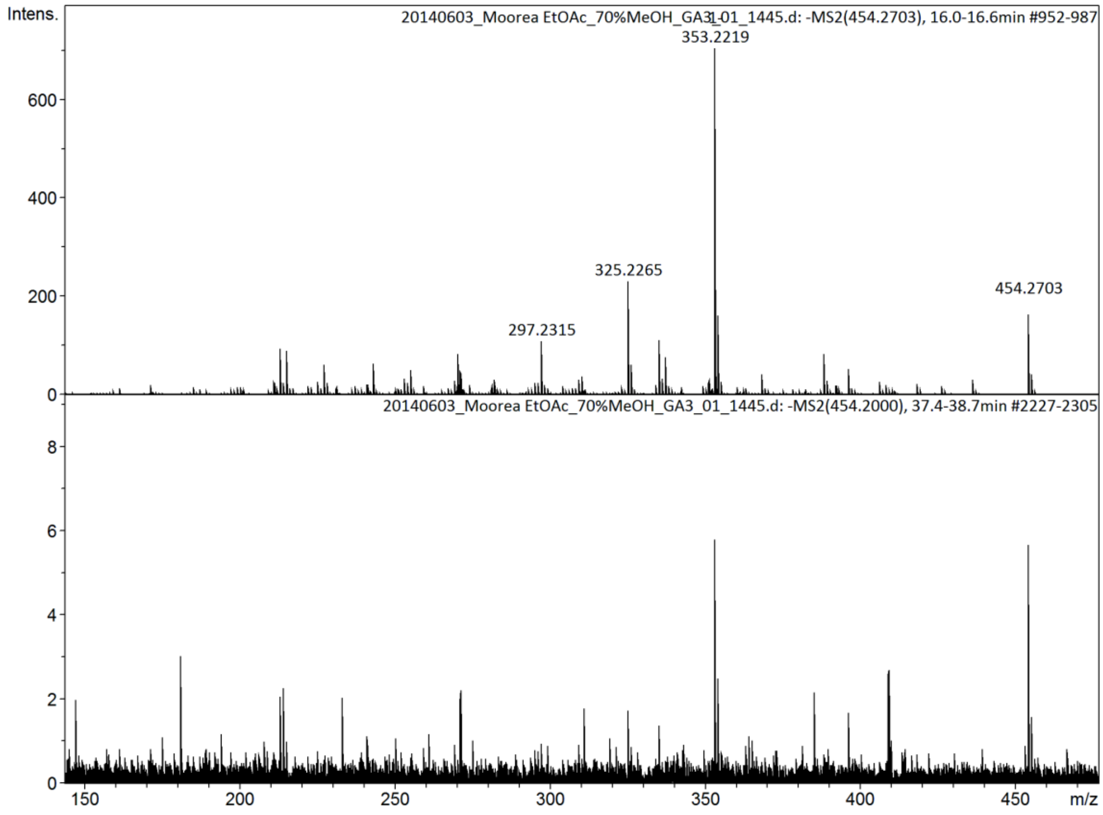

Figure S6. ^{1}H-NMR spectrum of **1**, in MeOD.

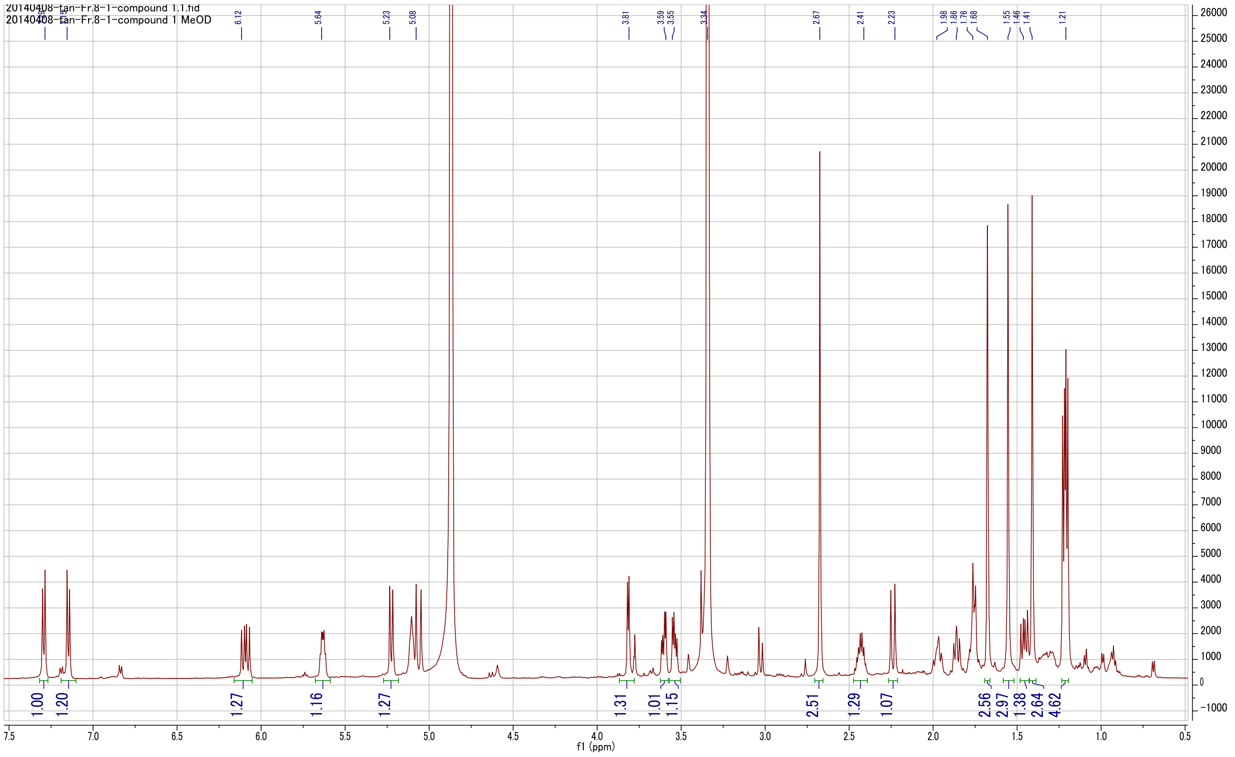

Figure S7. ^{13}C-NMR spectrum of **1**, in MeOD.

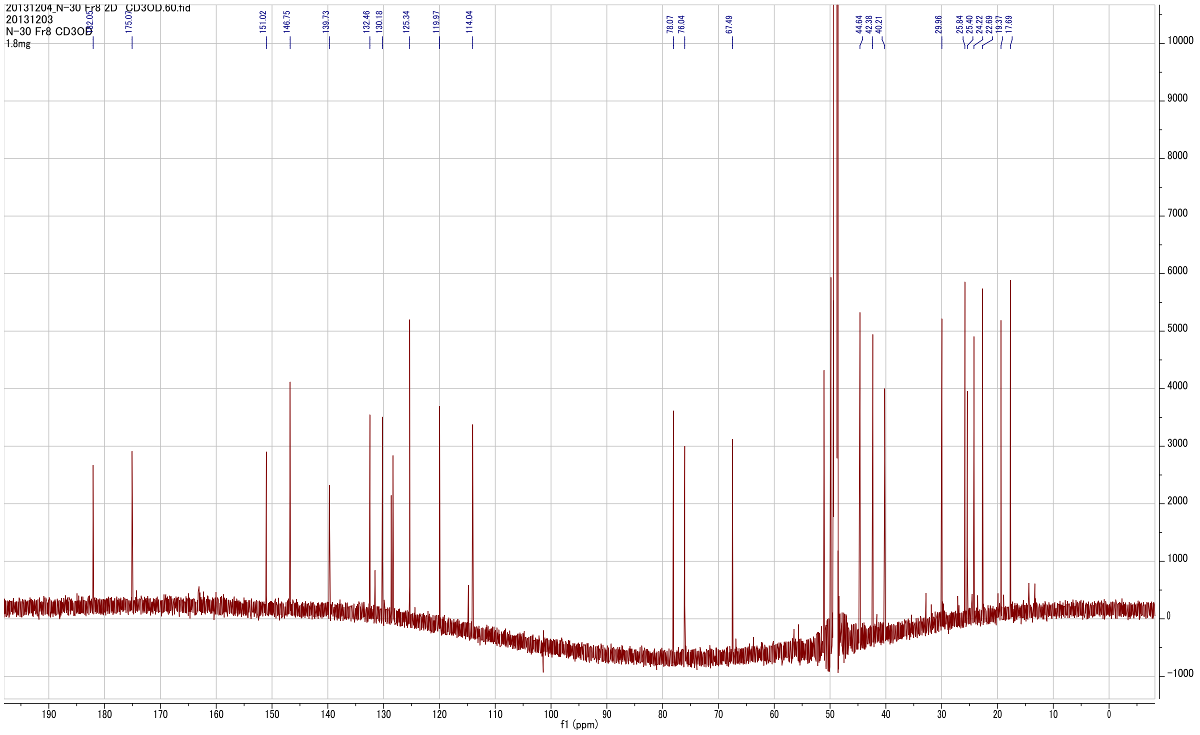

Figure S8. ^{1}H-^{1}H COSY spectrum of **1**, in MeOD.

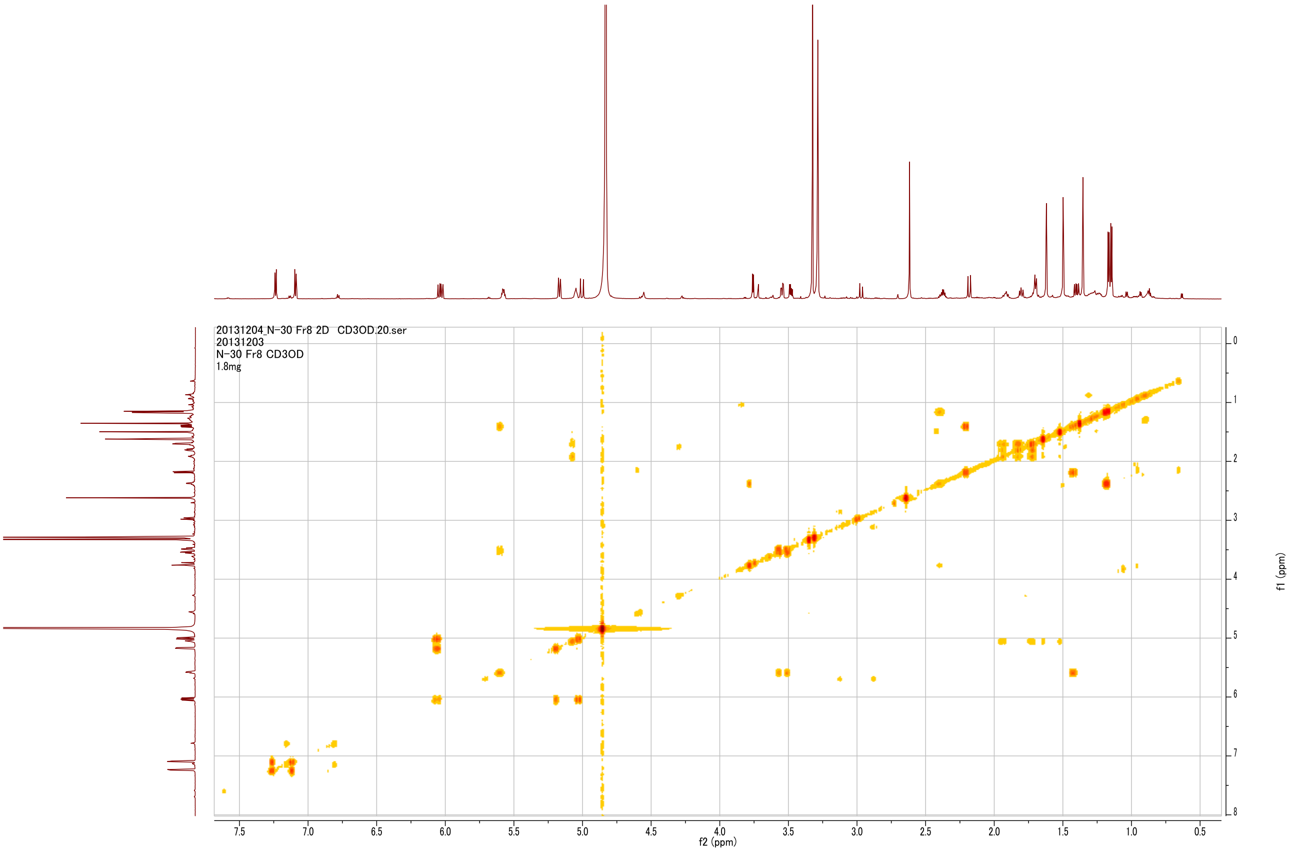

Figure S9. ^{1}H-^{13}C HSQC spectrum of **1**, in MeOD.

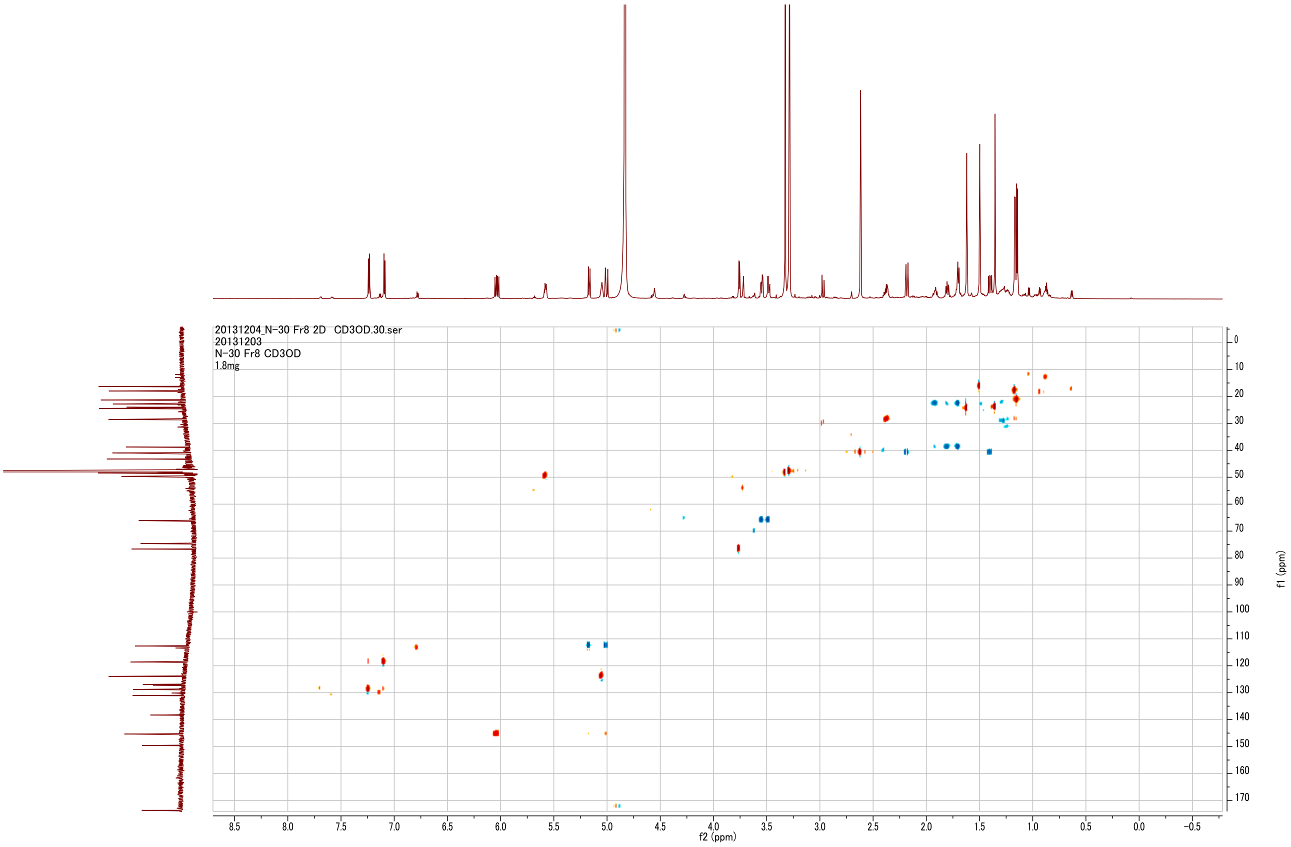

Figure S10. ^{1}H-^{13}C HMBC spectrum of **1**, in MeOD.

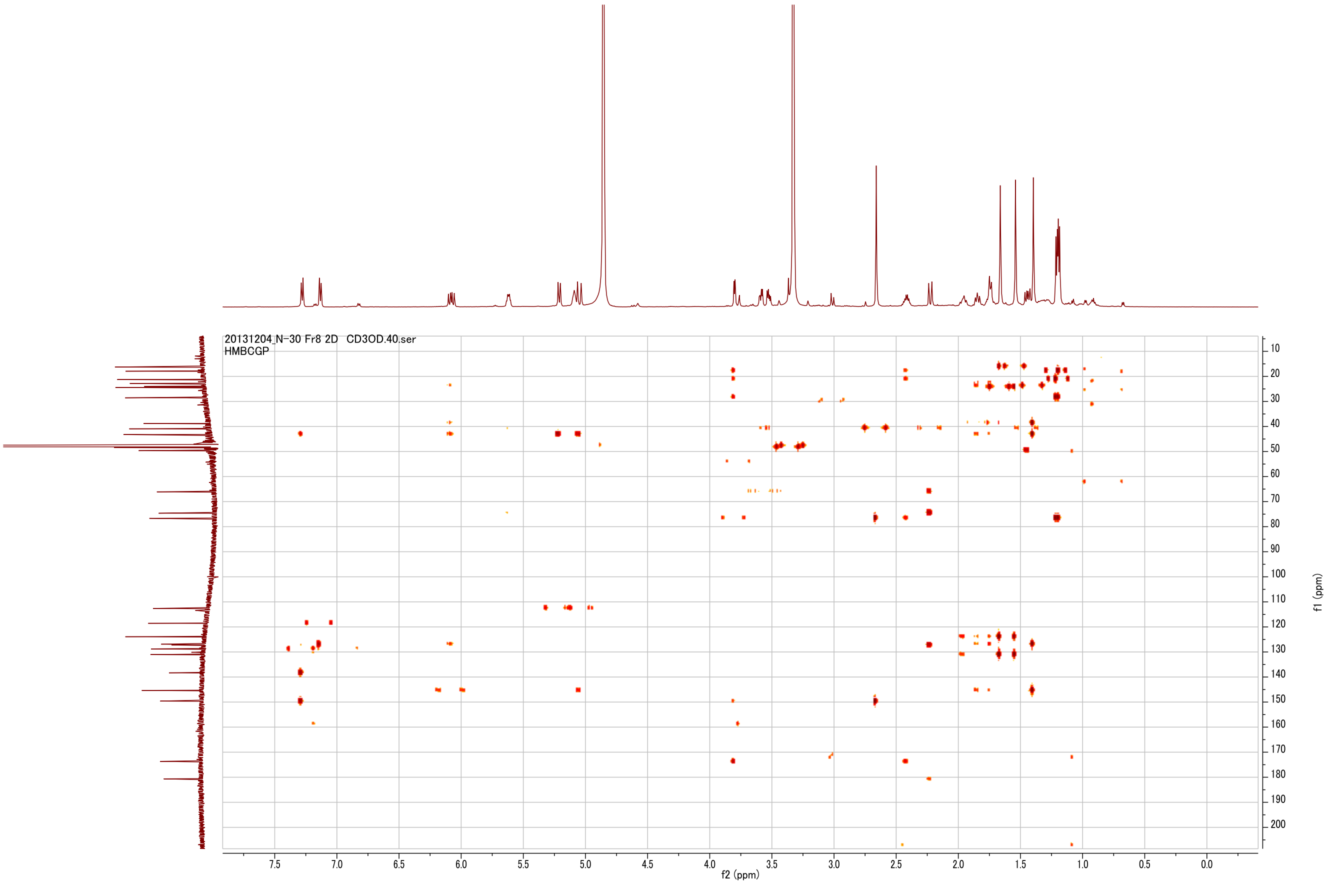

Figure S11. ^{1}H-^{1}H NOESY spectrum of **1**, in MeOD.

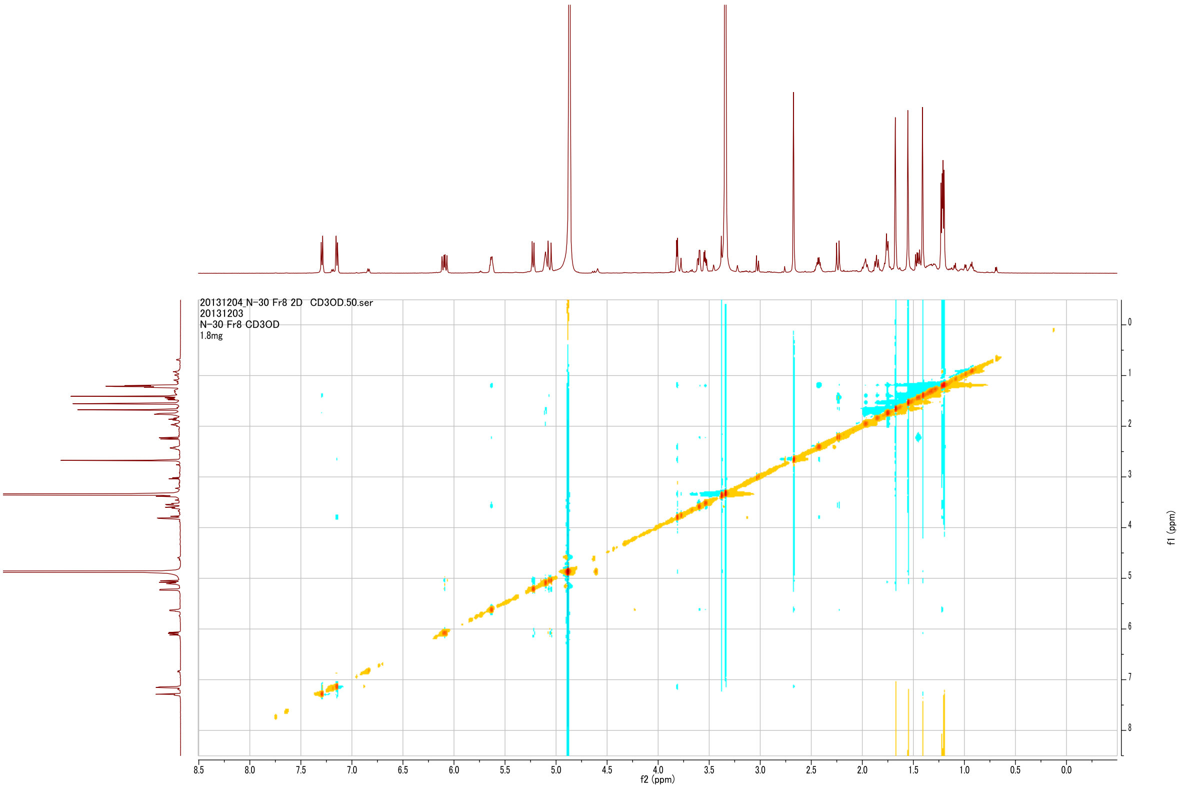

Figure S12. ^{1}H-NMR spectrum of **2**, in MeOD.

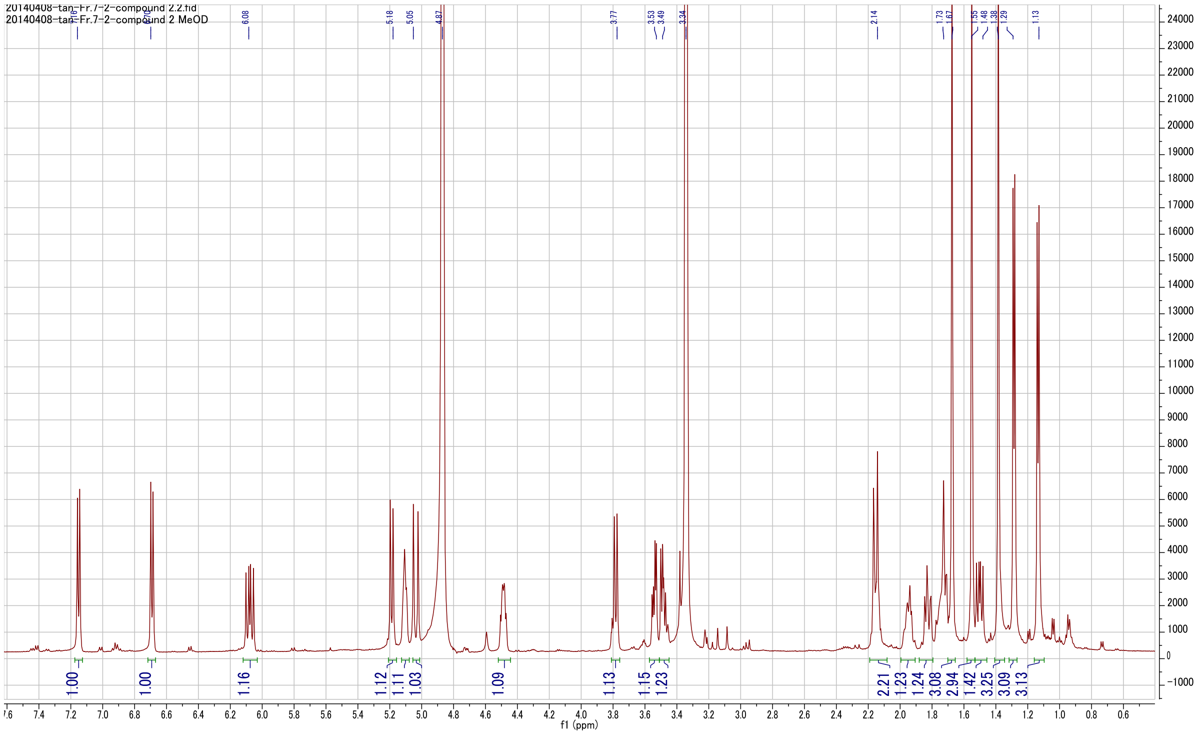

Figure S13. ^{13}C-NMR spectrum of **2**, in MeOD.

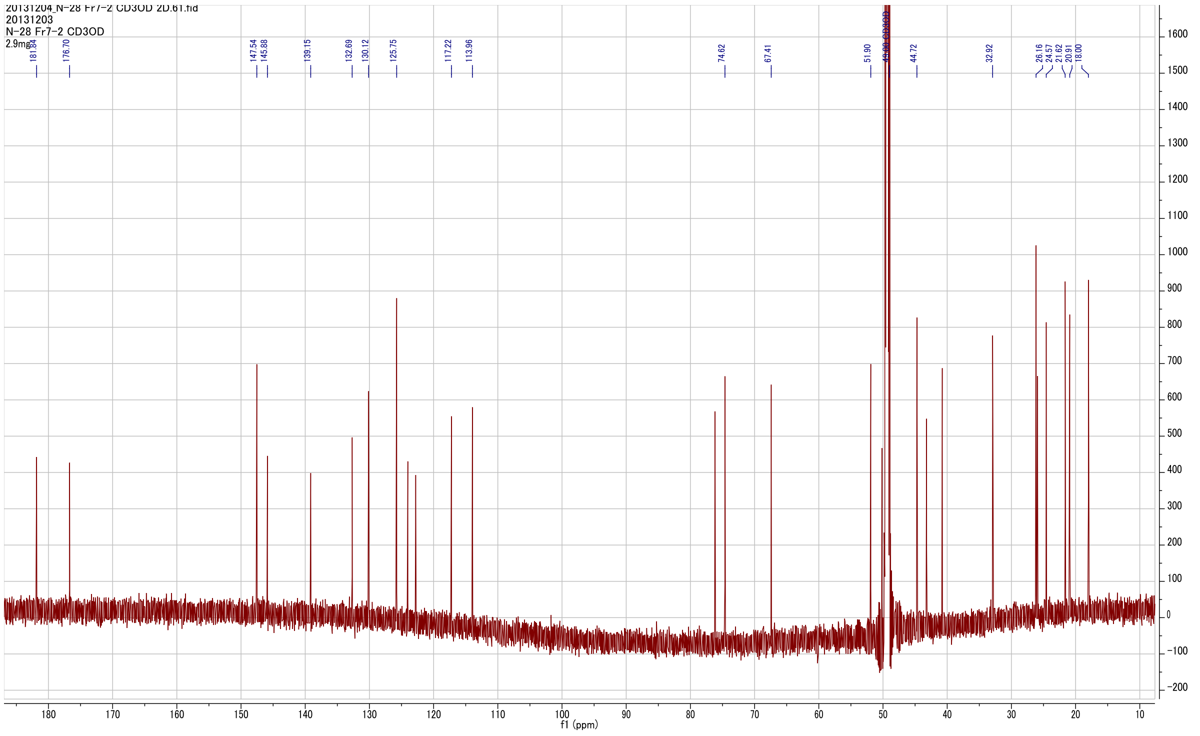

Figure S14. ^{1}H-^{1}H COSY spectrum of **2**, in MeOD.

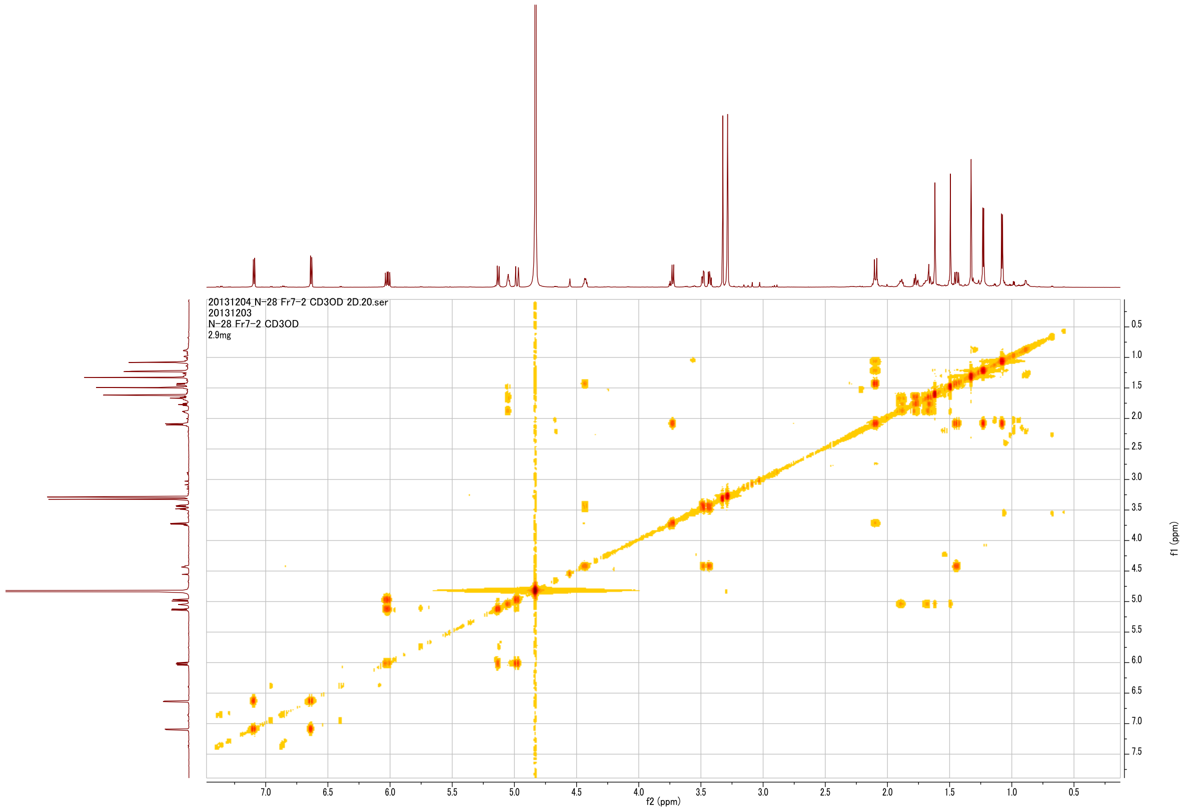

Figure S15. ^{1}H-^{13}C HSQC spectrum of **2**, in MeOD.

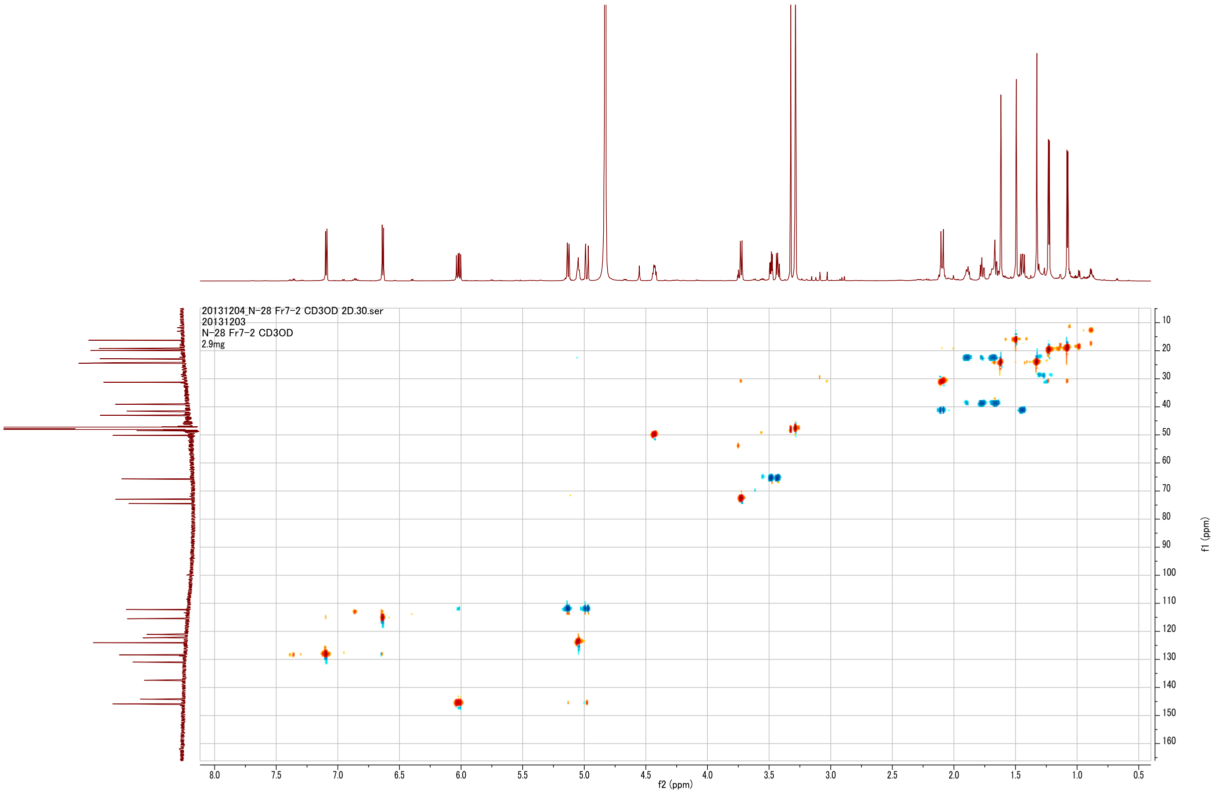

Figure S16. ^{1}H-^{13}C HMBC spectrum of **2**, in MeOD.

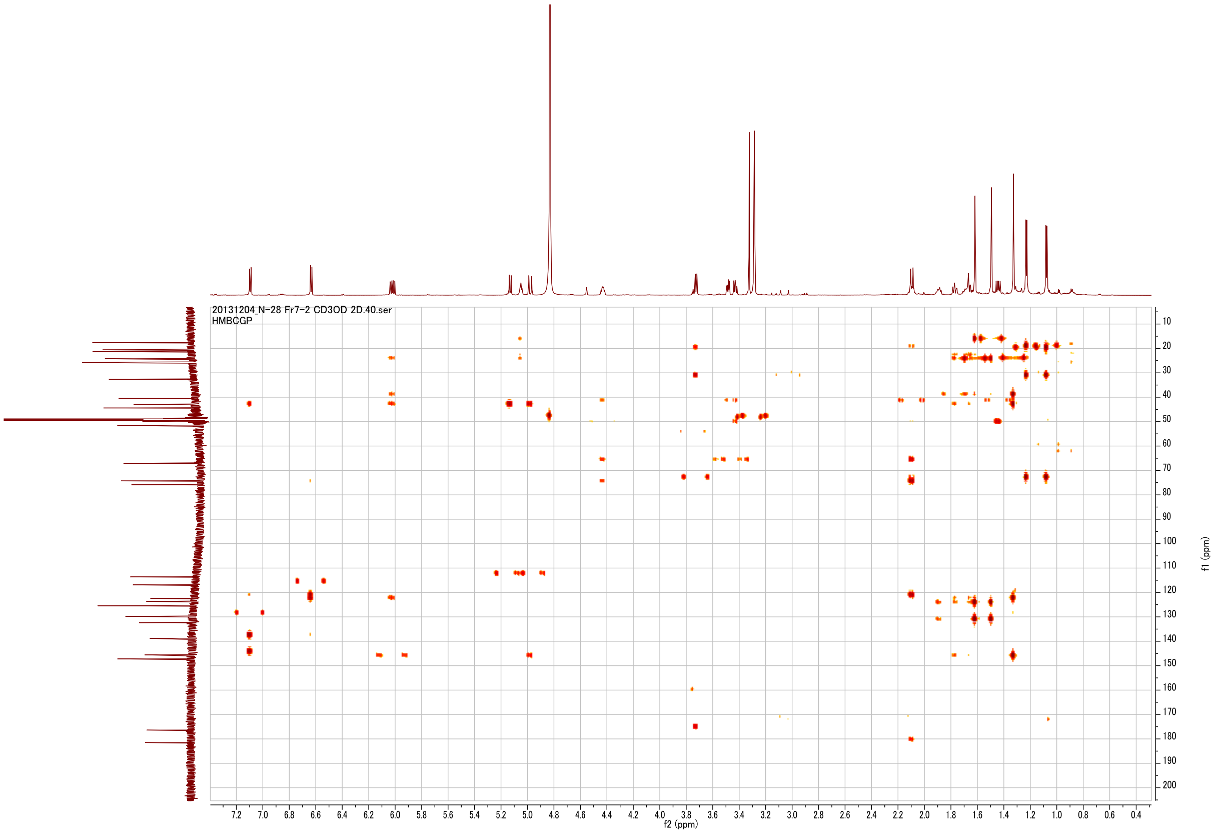

Figure S17. ^{1}H-^{1}H NOESY spectrum of **2**, in MeOD.

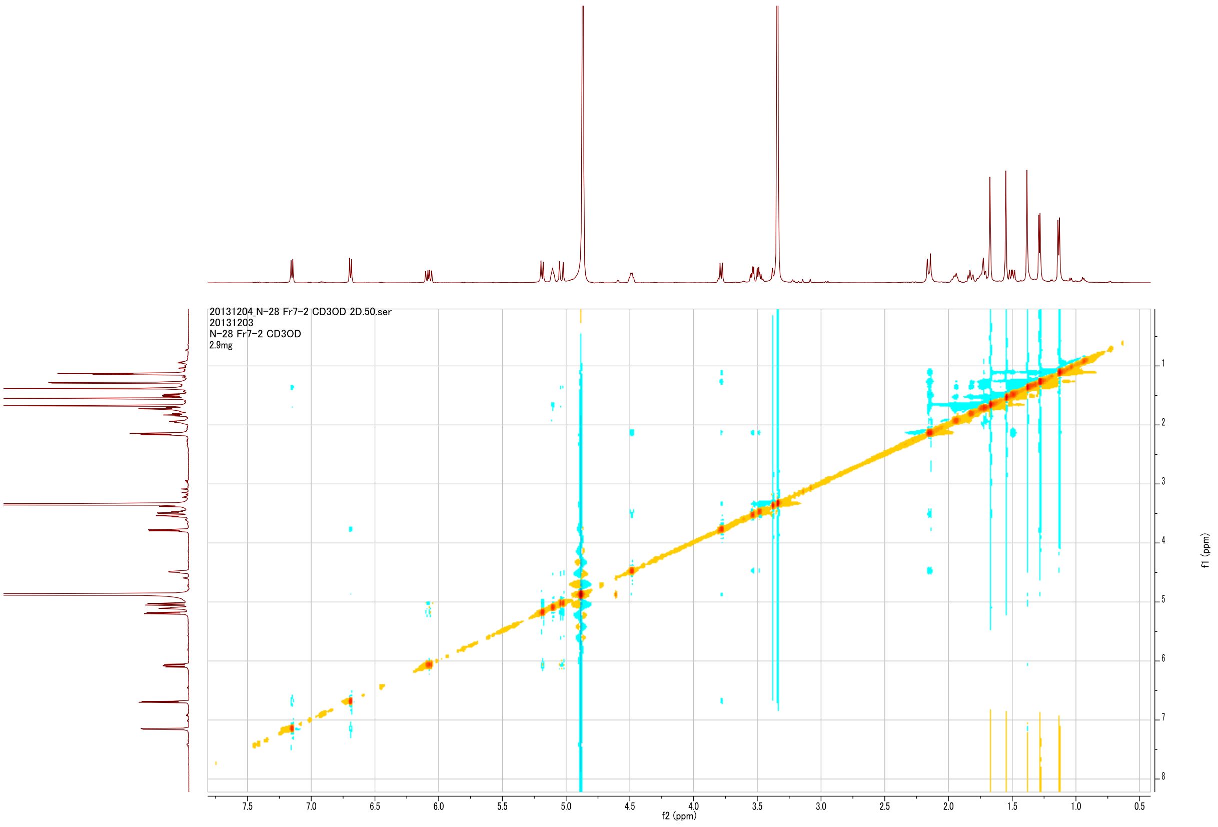

Figure S18. UV spectrum of **1** in EtOH.

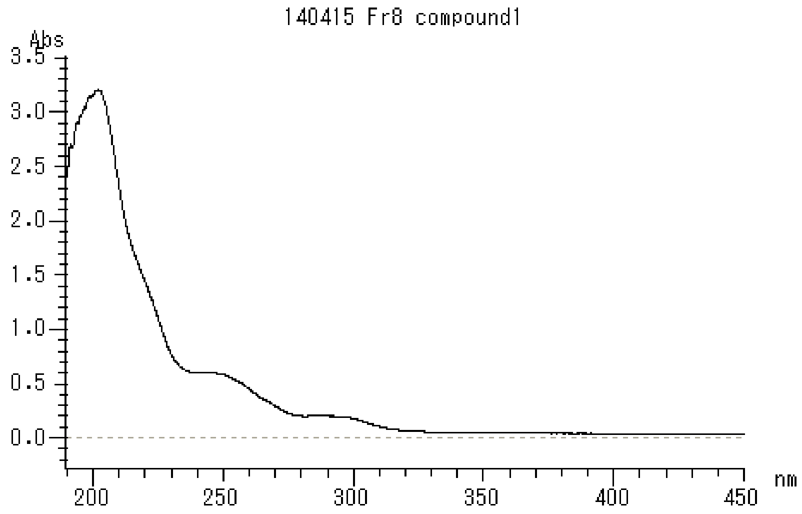

Figure S19. UV spectrum of **2** in EtOH.

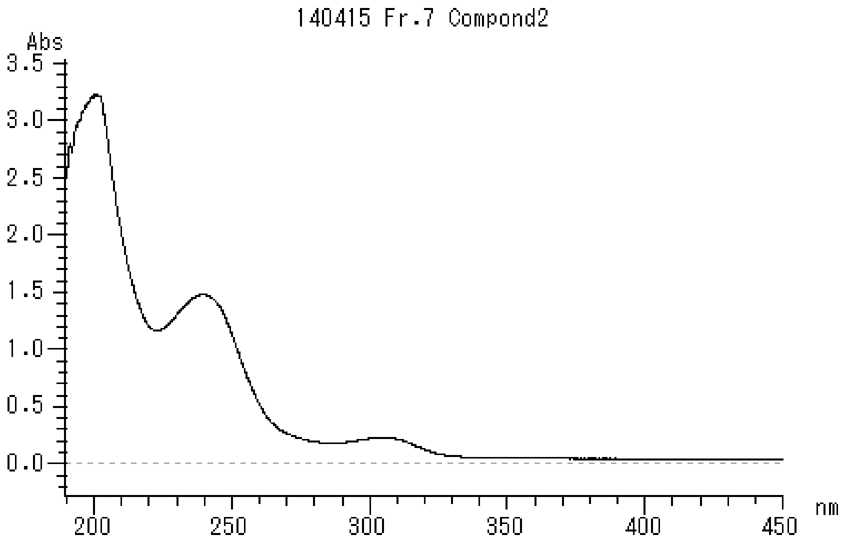

Figure S20. The structure of **2** and its key 2D NMR correlations in MeOD.

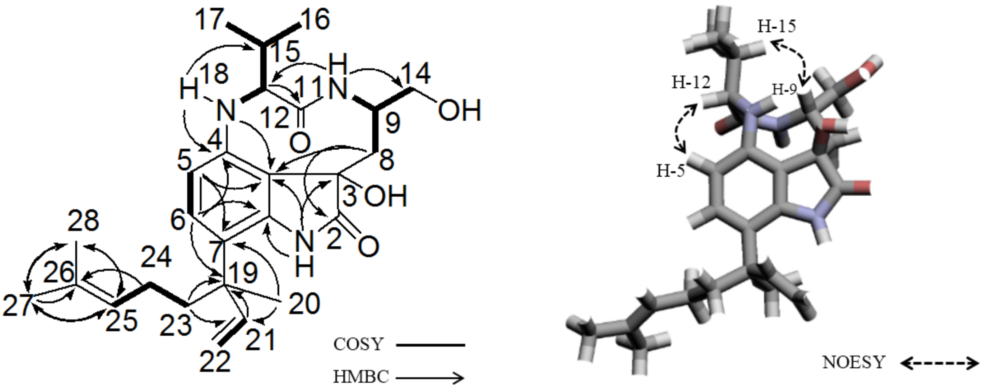

Figure S21. ^{1}H-NMR spectrum of **1**, in CDCl3.

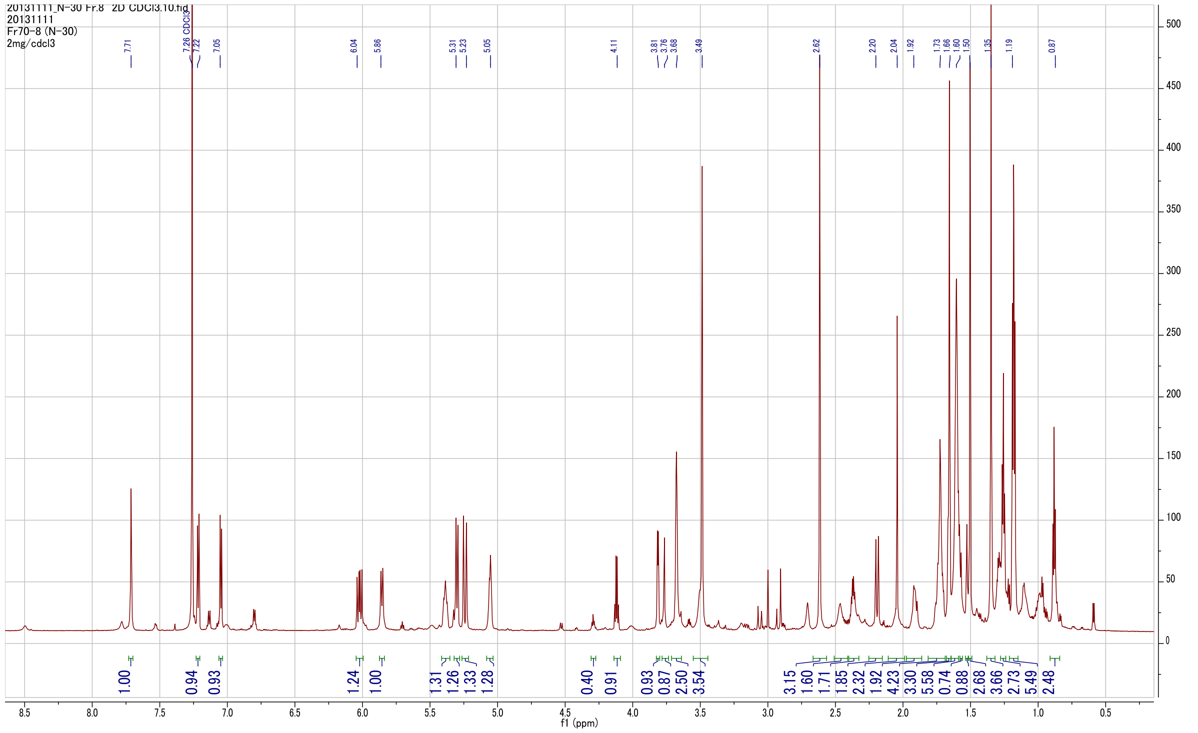

Figure S22. ^{13}C-NMR spectrum of **1**, in CDCl3.

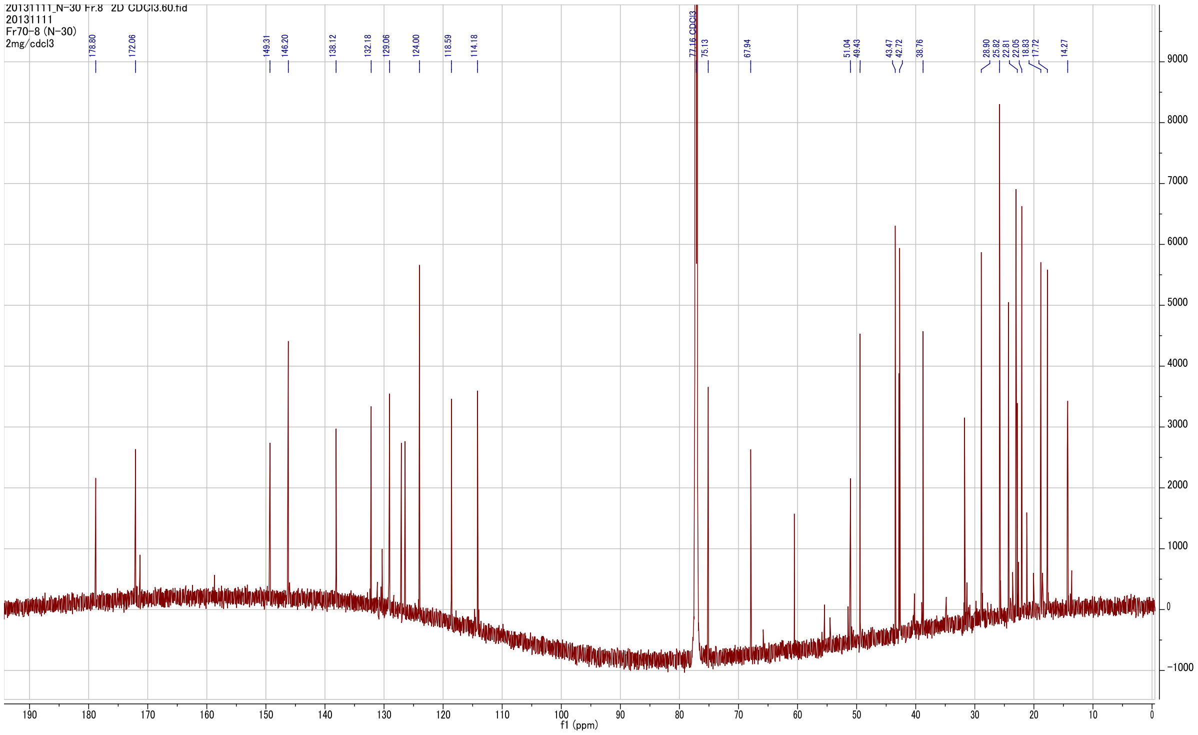

Figure S23. ^{1}H-NMR spectrum of **2**, in $CDCl_3$.

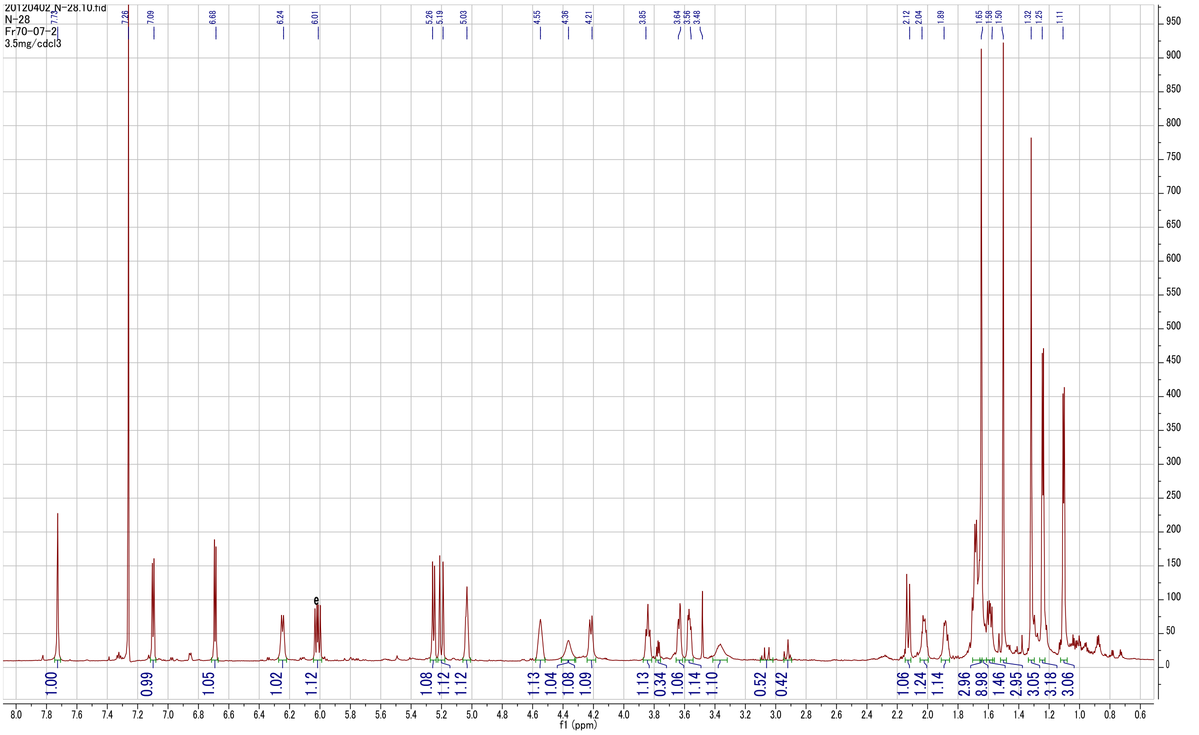

Figure S24. ^{13}C-NMR spectrum of **2**, in $CDCl_3$.

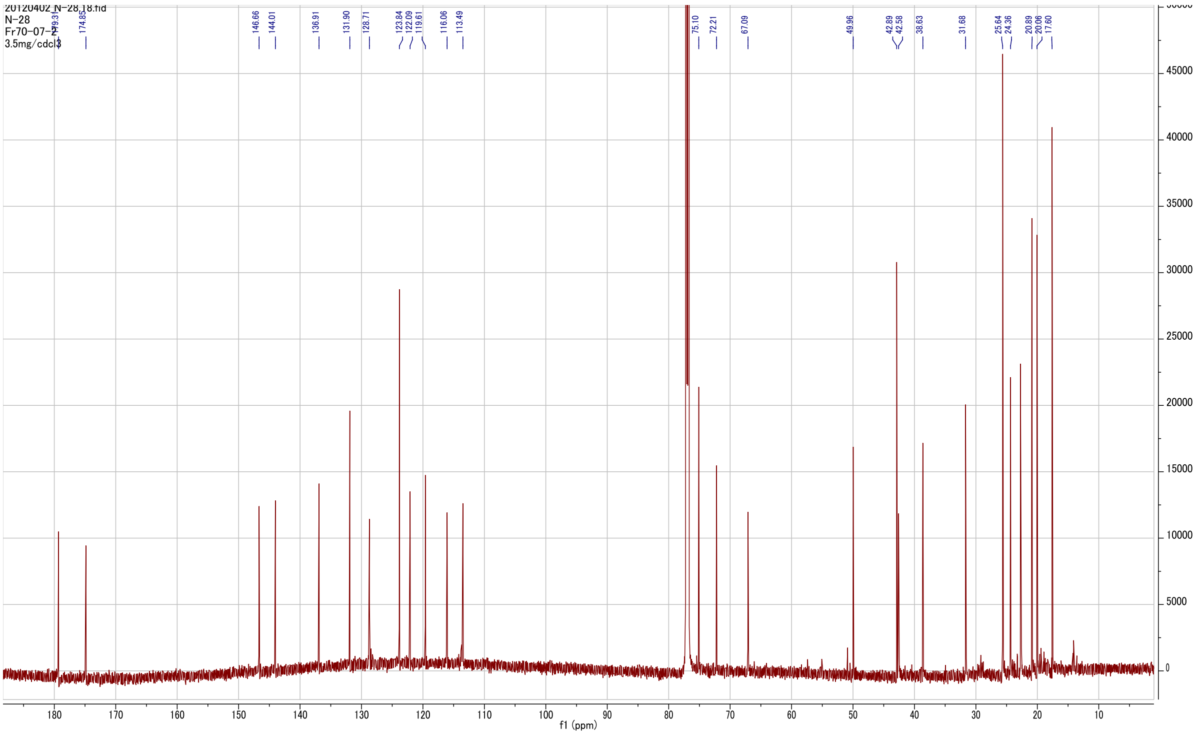

Figure S25. ^{1}H-^{1}H NOESY spectrum of **2**, in $CDCl_3$.

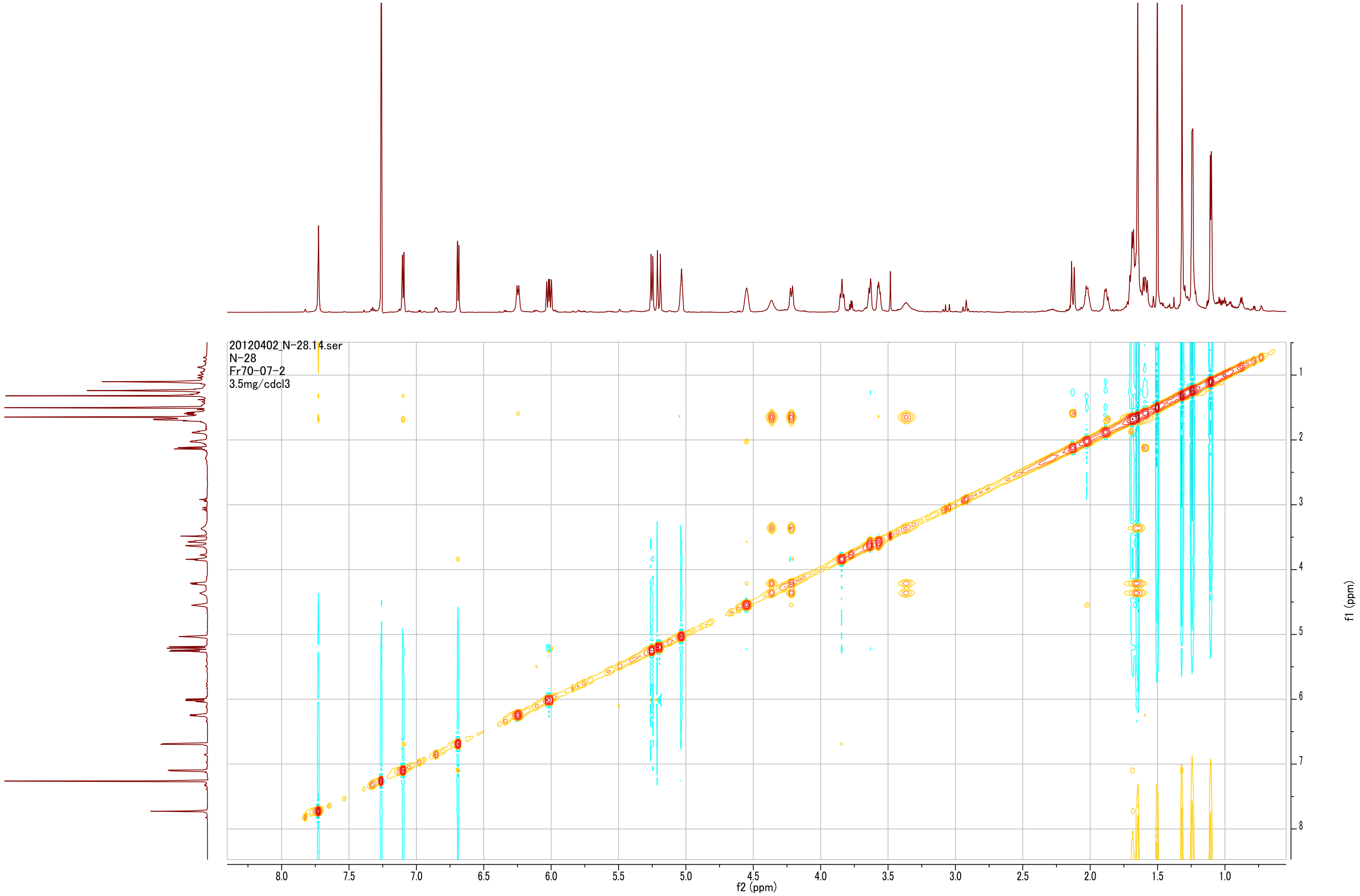

Figure S26. Key ^{1}H-^{1}H NOESY NMR correlations in $CDCl_3$.

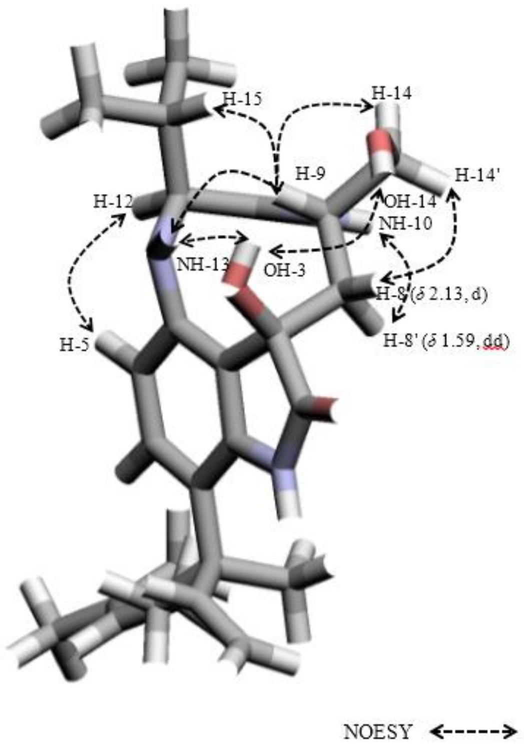

Figure S27. CD spectra of lyngbyatoxin A.

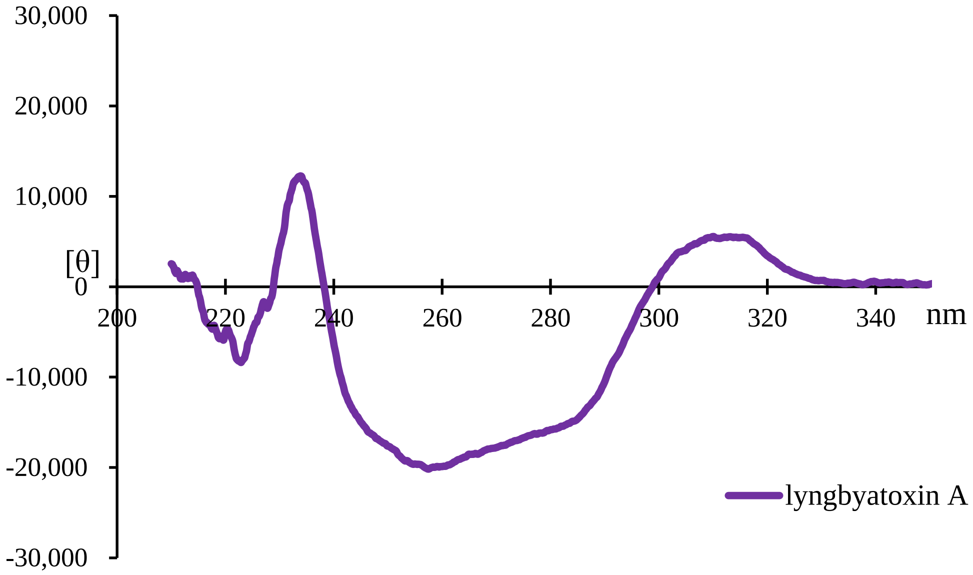

The CD spectral data were recorded in methanol with the concentration of 320 μmol/L, using a 2 mm path-length quartz cell. The measurements were performed at room temperature (25 ℃).

Table S1. ^{1}H and ^{13}C NMR chemical shifts observed for **1** and **2** in $CDCl_3$.

NO.	1		2	
	^{13}C	^{1}H, mult, *J* (Hz)	^{13}C	^{1}H, mult, *J* (Hz)
1		7.71, 1H, s		7.73, 1H, s
2	178.8		179.3	
3	75.2		75.1	
3a	126.4		119.6	
4	149.3		144.0	
5	118.6	7.05, 1H, d, *J* = 8.6	116.1	6.69, 1H, d, *J* = 8.6
6	129.1	7.22, 1H, d, *J* = 8.6	128.7	7.10, 1H, d, *J* = 8.6
7	127.1		122.1	
7a	138.1		136.9	
8	42.8	2.19, 1H, d, *J* = 15.1	42.9	2.13, 1H, d, *J* = 15.6
		1.50, 1H, d, *J* = 15.1		1.59, 1H, dd, *J* = 9.3, 14.9
9	49.4	5.39, 1H, m	50.0	4.55, 1H, m
10		5.86, 1H, d, *J* = 11.1		6.25, 1H, d, *J* = 10.7
11	172.1		174.9	
12	77.1	3.81, 1H, d, *J* = 5.2	72.2	3.84, 1H, t, *J* = 10.4
13				4.22, 1H, d, *J* = 12.5
14	67.9	3.68, 2H, s	67.1	3.64, 1H, d, *J* = 10.5
				3.57, 1H, dd, *J* = 5.7, 10.5
15	28.9	2.37, 1H, m	31.7	2.02, 1H, m
16	22.0	1.18, 3H, d, *J* = 7.4	20.9	1.24, 3H, d, *J* = 6.5
17	18.8	1.18, 3H, d, *J* = 6.9	20.1	1.10, 3H, d, *J* = 6.5
18	42.7	2.62, 3H, s		
19	43.5		42.6	
20	24.3	1.35, 3H, s	24.4	1.32, 3H, s
21	146.2	6.02, 1H, dd, *J* = 10.7, 17.4	146.7	6.02, 1H, dd, *J* = 10.8, 17.7
22	114.2	5.30, 1H, d, *J* = 10.7	113.5	5.25, 1H, d, *J* = 10.8
		5.24, 1H, d, *J* = 17.7		5.20, 1H, d, *J* = 17.7
23	38.7	1.73, 2H, m	38.6	1.69, 2H, m
24	23.1	1.73, 1H, m	22.8	1.69, 1H, m
		1.92, 1H, m		1.88, 1H, m
25	124.0	5.05, 1H, m	123.8	5.03, 1H, m
26	132.2		131.9	
27	25.8	1.66, 3H, s	25.6	1.65, 3H, s
28	17.7	1.50, 3H, s	17.6	1.50, 3H, s
OH on 3		3.49, 1H, s		4.36, 1H, br s
OH on 14		2.46, 1H, br s		3.37, 1H, m

s, singlet; d, doublet; t, triplet; m, multiplet.

© 2014 by the authors; licensee MDPI, Basel, Switzerland. This article is an open access article distributed under the terms and conditions of the Creative Commons Attribution license (http://creativecommons.org/licenses/by/4.0/).
